# Facile synthesis of hydrophilic magnetic graphene nanocomposites via dopamine self-polymerization and Michael addition for selective enrichment of N-linked glycopeptides

**DOI:** 10.1038/s41598-019-56944-4

**Published:** 2020-01-09

**Authors:** Changfen Bi, Ye Yuan, Yuran Tu, Jiahui Wu, Yulu Liang, Yiliang Li, Xiwen He, Langxing Chen, Yukui Zhang

**Affiliations:** 1 Tianjin Key Laboratory of Radiation Medicine and Molecular Nuclear Medicine, Institute of Radiation Medicine, Peking Union Medical College & Chinese Academy of Medical Sciences, Tianjin, 300192 China; 2 College of Chemistry, Tianjin Key Laboratory of Biosensing and Molecular Recognition, State Key Laboratory of Medicinal Chemical Biology, Nankai University, Tianjin, 300071 China; 3 Collaborative Innovation Center of Chemical Science and Engineering (Tianjin), Tianjin, 300071 China; 4 Dalian Institute of Chemical Physics, Chinese Academy of Sciences, Dalian, 116023 China

**Keywords:** Chemical biology, Biomarkers, Chemistry, Materials science

## Abstract

The development of methods to effectively capture N-glycopeptides from the complex biological samples is crucial to N-glycoproteome profiling. Herein, the hydrophilic chitosan–functionalized magnetic graphene nanocomposites (denoted as Fe_3_O_4_-GO@PDA-Chitosan) were designed and synthesized via a simple two-step modification (dopamine self-polymerization and Michael addition). The Fe_3_O_4_-GO@PDA-Chitosan nanocomposites exhibited good performances with low detection limit (0.4 fmol·μL^−1^), good selectivity (mixture of bovine serum albumin and horseradish peroxidase tryptic digests at a molar ration of 10:1), good repeatability (4 times), high binding capacity (75 mg·g^−1^). Moreover, Fe_3_O_4_-GO@PDA-Chitosan nanocomposites were further utilized to selectively enrich glycopeptides from human renal mesangial cell (HRMC, 200 μg) tryptic digest, and 393 N-linked glycopeptides, representing 195 different glycoproteins and 458 glycosylation sites were identified. This study provides a feasible strategy for the surface functionalized novel materials for isolation and enrichment of N-glycopeptides.

## Introduction

Protein N-glycosylation, as one of the most prevalent post-translational modifications (PTMs), can significantly change the structure, increase stability and subsequently endow new functions of protein^1–3^. Meanwhile, this modification plays determinant roles in physiological processes, such as molecular and cell recognition, signal transduction, immune response. Aberrant protein N-glycosylation is associated with human disease, such as rheumatoid arthritis, lupus erythematosus, Alzheimer’s disease (AD) and so on^4–6^ Therefore, the thorough analysis of protein N-glycosylation is beneficial for elucidating pathogenesis of many diseases and for the discovery of new clinical biomarkers and effective disease control^7^. In the recent years, mass spectrometry (MS) has been widely used in N-glycoproteme analysis due to its high sensitivity and high-throughput and capacity to analyze disease-associated glycoforms^8^. However, the low abundance of N-glycoproteins in biological samples, poor ionization of N-glycopeptides and the heterogeneity of glycan structures make the direct MS analysis extremely difficult^8^. Therefore, an enrichment step of N-glycopeptides prior to MS analysis is a prerequisite for the successful glycoproteomics analysis.

A variety of enrichment strategies towards glycoproteins/glycopeptides have been developed, such as boronic acid chemistry^9–12^, immobilized metal ion affinity chromatography (IMAC)^13–15^, molecularly imprinting method^16–18^, hydrophilic interaction liquid chromatography (HILIC)^19–21^ and so on. Among them, owning to good MS compatibility, excellent reproducibility, unbiased enrichment performance and simple operating process, HILIC approach, based on the hydrophilicity differences between glycopeptides and non-glycopeptides, is widely adopted in glycopeptides enrichment. Chitosan is a cationic polymer obtained by deacetylation of chitin, and has gained more attention as drug delivery carriers owning to its bio-safety, biocompatibility and biodegradability^22^. The polar groups (-OH, -NH_2_) endowed chitosan with selectivity towards glycopeptides through hydrogen bond between the glycan moieties and -OH/-NH_2_. Chitosan microspheres as adsorbent for isolation need an inconvenient and time-consuming centrifugation separation^23^. In recent years, magnetic separation has received extensive attention because of its better separation efficiency in contrast to the traditional approach. The combination of HILIC enrichment strategy and magnetic separation technology realized the rapid and efficient enrichment and separation of glycoproteins/glycopeptides. Li *et al*. fabricated Fe_3_O_4_@G6P magnetic microspheres for specific capture of N-linked glycopeptides^24^. The hydrophilic glucose-6-phosphate immobilized on the Fe_3_O_4_ nanoparticles endowed the microspheres with high selectivity and sensitivity. Fang *et al*. modified chitosan on magnetic Fe_3_O_4_ nanoparticles^25^. The HILIC microspheres showed the strong ability of fast magnetic separation and recognition toward glycopeptides. Notwithstanding these excellent reports, the synthesis of novel magnetic materials is still attracting extensive attentions aimed at optimizing the enrichment selectivity and sensitivity towards glycoproteins/glycopeptides. Magnetic Fe_3_O_4_ nanoparticle-decorated GO (Fe_3_O_4_-GO) has magnetic responsibility and large surface area, and has been widely used for glycoproteins/glycopeptides enrichment^26,27^. Although some efficient strategies (self-assembly, Au-S bond) were applied to fabricate hydrophilic materials, they suffered from tedious operation^28,29^. Based on these, the assembly of chitosan coated Fe_3_O_4_-GO nanocomposites by a convenient Michael addition reaction would be very attractive.

**Figure 1:**
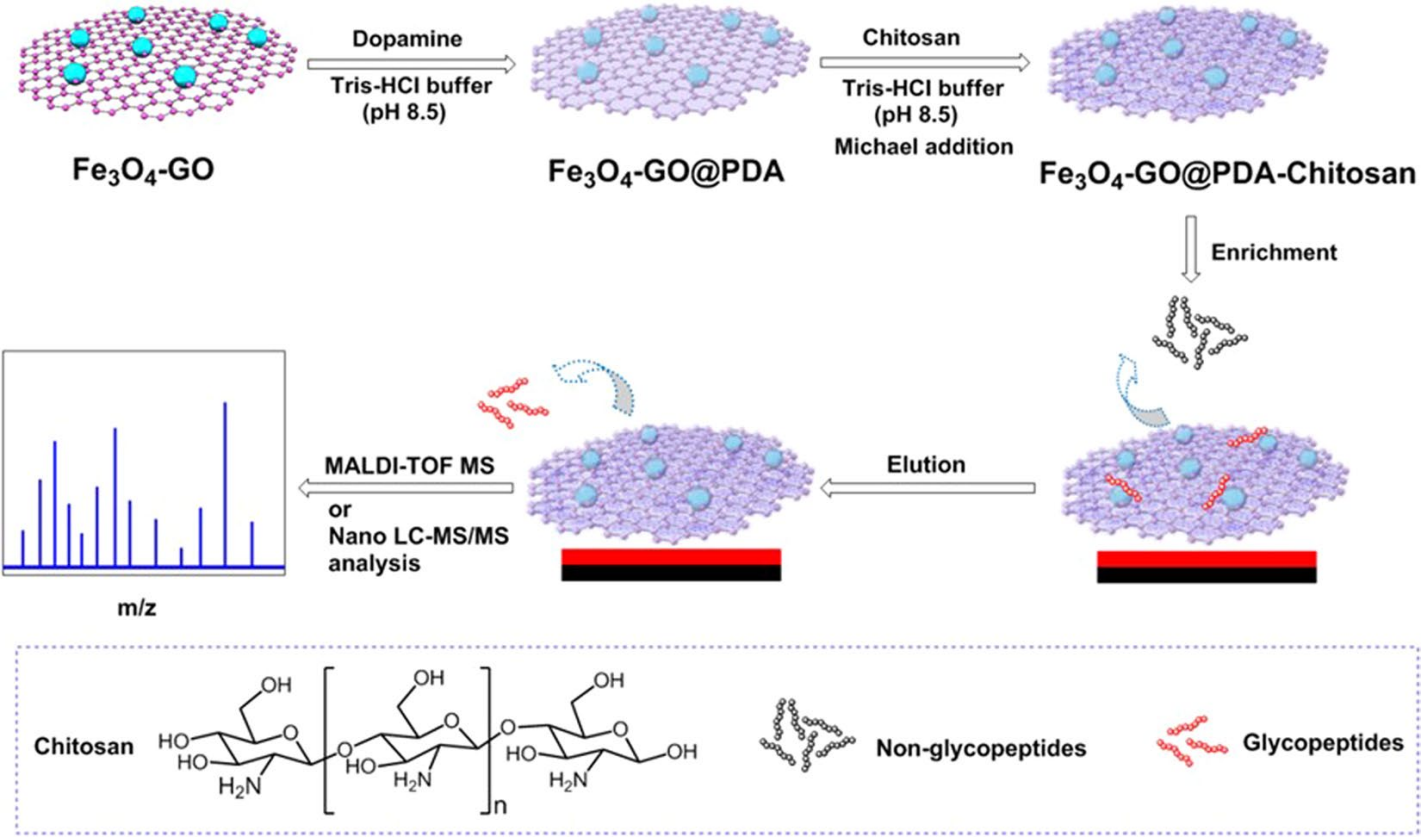
Schematic illustration of the procedure for preparation of Fe_3_O_4_-GO@PDAand Fe_3_O_4_-GO@PDA-Chitosan and N-glycopeptides enrichment.

Herein, a new type of chitosan-functionalized hydrophilic magnetic nanocomposites, Fe_3_O_4_-GO@PDA-Chitosan (Fig. 1) was assembled via dopamine self-polymerization and Michael addition. Briefly, Fe_3_O_4_ NPs were interspersed on the surface of graphene oxide nanocomposites by solvothermal reaction. Polydopamine (PDA)-coated magnetic graphene was prepared via dopamine self-polymerization. Chitosan was grafted on the surface of Fe_3_O_4_-GO@PDA nanocomposites via Michael addition. The dopamine self-polymerization and Michael addition reaction occurred simply under mechanical agitation in Tris-HCl buffer. The polydopamine layer and chitosan on the surface of magnetic graphene could effectively enhance hydrophilic enrichment performance of magnetic nanocomposites for N-glycopeptides from the complex biological samples under an external magnetic field. This novel nanocomposite was applied to achieve good selectivity (mixture of bovine serum albumin and horseradish tryptic digests at a molar ration of 10:1), sensitivity (0.4 fmol·μL^−1^), binding capacity (75 mg·g^−1^) for N-glycopeptides enrichment.

## Experimental Section

### Materials

Chitosan (with a deacetylation, degree of 98%) was purchased from Aladdin (Shanghai, China). Horseradish peroxidase (HRP), immunoglobulin G (IgG), peptide-N4-(N-acetyl-β-D-glucosaminyl) asparagine amidase F (PNGase F), bovine serum albumin (BSA), and HPLC-grade acetonitrile (ACN) were purchased from Sigma-Aldrich (USA) (Beijing, China). Dithiothreitol (DTT), urea, ammonium bicarbonate (NH_4_HCO_3_) and iodoacetamide (IAA) were obtained purchased from Solarbio (China). Trifluoroacetic acid (TFA) was purchased from J&K (Beijing, China). 2,5-Dihydroxybenzoic acid (DHB) was obtained from TCI (Japan) (Shanghai, China). Trypsin was from Sangon Biotech Co. Ltd. (Shanghai, China). Graphene oxide (GO) was purchase from XF NANO (Nanjing, China). Dopamine chloride and tris(hydroxymethyl)aminomethane (Tris) were obtained from Tianjin Heowns Biotech Co. Ltd. (Tianjin, China). Iron (III) chloride hexahydrate (FeCl_3_·6H_2_O), sodium acetate (NaAc), ethylene glycol (EG), acetic acid, hydrogen peroxide (H_2_O_2_), ethanol, methanol were purchased from Concord Chemical Ltd. (Tianjin, China). Deionized water (18.25 MΩ cm) was prepared with a Milli-Q water purification system (Millipore, Milford, MA, USA).

### Characterization

The morphology, structure and performance of the synthesized nanocomposites were evaluated according to a previous report^30^. Transmission electron microscope (TEM) images were obtained on a JEM-2100F (Japan) transmission electron microscope. Fourier transform infrared (FT-IR) spectra (4000-400 cm^−1^) in KBr were recorded using the BRUKER TENSOR 27 Fourier transform infrared spectrophotometer. The crystal structure of the nanocomposites was performed on a Rigaku (Japan) D/max/2500 v/pc with nickel-filtered Cu Kα source. The XRD patterns were collected in the range 3 < 2θ < 80° at a scan rate of 4.0°/min. The X-ray photoelectron spectra were obtained on a Thermo Fischer (USA) ESCALAB 250Xi X-ray photoelectron spectrometer (XPS) with an Mg Kα anode (15 kV, 400 W) at a takeoff angle of 45°. The source X-rays were not filtered and the instrument was calibrated against the C 1 s band at 285 eV. The magnetic properties were analyzed with a LDJ9600-1 (USA) vibrating sample magnetometer (VSM). The hydrophilicity of the nanocomposites was revealed with contact angle analyzer JY-82B (Dingsheng, China). Zeta potential of the nanocomposites was measured by Brook haven ZETAPALS/BI-200SM (USA) at room temperature. Thermogravimetric analysis (TGA) was carried out in nitrogen atmosphere at a heating rate of 10 °C·min^−1^ from room temperature to 800 °C (NETZCHSTA 449 F3, Germany). Matrix assisted laser desorption/ionization time-of-flight mass spectrometry (MALDI-TOF MS) were performed on a Bruker Auto flex III LRF200-CID instrument (Bruker Daltonics, Germany) with a pulsed nitrogen laser operating at 337 nm in linear positive ion mode and DHB (25 mg·mL^−1^, ACN/H_2_O/TFA, 80:19:1, v/v/v) was used as matrix.

### Preparation of Fe_3_O_4_-GO nanocomposites

Fe_3_O_4_-GO NPs were synthesized with a solvothermalmethod^29^. Typically, GO (48.7 mg), NaAc (1.36 g) and FeCl_3_·6H_2_O (152 mg) were dissolved in EG (30 mL) under sonication for 2 h. The homogeneous solution was poured into a Teflon-lined stainless-steel autoclave (50 mL) and maintained at 180 °C for 16 h. With the help of an external magnetic field, the prepared Fe_3_O_4_-GO nanocomposites were separated from the reaction solvent and washed with deionized water and ethanol for several times, and dried in vacuum at 40 °C for 3 h.

### Preparation of Fe_3_O_4_-GO@PDA nanocomposites

The Fe_3_O_4_-GO nanocomposites (40 mg) were dispersed in Tris buffer (100 mL, 10 mM, pH 8.5) under sonication, and dopamine chloride (30 mg) was added to the above solution. The mixture was stirred mechanically at 60 °C for 24 h. In an external magnetic field, the prepared Fe_3_O_4_-GO@PDA nanocomposites were separated from the reaction solvent and washed with deionized water and ethanol for several times, and dried in vacuum at 40 °C for 3 h.

### Degradation of chitosan by hydrogen peroxide

5% H_2_O_2_ (100 mL) was added dropwise into the solution of Chitosan (10 g) in 2% acetic acid (200 mL). The mixture was stirred magnetically at 60 °C for 8 h. Then the reaction solution was cooled and passed through two layers of filter paper. The aqueous phase obtained was retained and the pH was adjusted to 10.0 with 1 N NaOH, set aside for 2 h, and again filtrated through two layers of filter paper. After concentration under reduced pressure, the solution was diluted with methanol, set aside for 5 h. The white precipitation was collected via filtration and washed with methanol. The white powder was obtained after drying under vacuum.

### Preparation of Fe_3_O_4_-GO@PDA-Chitosan nanocomposites

Into the solution of chitosan (120 mg) in Tris-HCl buffer (120 mL, pH 8.5), the Fe_3_O_4_-GO@PDA-Chitosan nanocomposites (30 mg) was dispersed under sonication. The mixture was stirred mechanically at room temperature for 24 h. In an external magnetic field, the prepared Fe_3_O_4_-GO@PDA-Chitosan nanocomposites were separated from the reaction solvent and washed with deionized water and ethanol for several times, and dried in vacuum at 40 °C for 3 h.

### Digestion of proteins

1 mg HRP (human IgG or BSA) was dissolved in 1 mL NH_4_HCO_3_ solution (50 mM, pH 8.3) under sonication and denatured by boiling for 15 min in water bath. And then, the denatured proteins were reduced with 3.1 mg DTT at 37 °C for 2 h in water bath and alkylated by 7.2 mg IAA using a shaking table at room temperature in the dark for 40 min. The mixture was incubated with trypsin at an enzyme-protein ratio of 1:25 (w/w) at 37 °C for 16 h. The tryptic digests were stored at −20 °C for later use.

Proteins extracted from human renal mesangial cells (HRMC) were precipitated by trichloroacetic acid and collected by centrifugation. The pellet was dissolved in 100 mM of NH_4_HCO_3_. The proteins underwent reduction, alkylation, and enzymolysis in sequence. The resulting digests were desalted using Sep-pak C18 cartridges (Water Ltd., Elstree, UK), evaporated to dryness, and stored at −20 °C for later use.

### N-Glycopeptides enrichment under hydrophilic mode

Fe_3_O_4_-GO@PDA-Chitosan nancomposites (or Fe_3_O_4_-GO@PDA, 40 μg) were rinsed thrice with the loading buffer (ACN/H_2_O/TFA = 89:10.5:0.5, v/v/v), and then dispersed via ultrasonication in the above buffer (400 μL) containing a determined amount of HRP or IgG digests. After incubation at room temperature for 30 min, the nanocomposites were then washed thrice with the loading buffer. Finally, the N-glycopeptides captured by the Fe_3_O_4_-GO@PDA-Chitosan nancomposites (or Fe_3_O_4_-GO@PDA) were released by 2 × 13 μL elution buffer (ACN/H_2_O/TFA = 30:69.9:0.1, v/v/v). The supernatant was collected, lyophilized, re-dissolved in 4 μL elution buffer and analyzed by MALDI-TOF MS.

For the enrichment of N-glycopeptides from digests of human renal mesangial cells (HRMC, kindly donated by Dr. Mingzhen Li (Metabolic Diseases Hospital, Tianjin Medical University, Tianjin, China)), the operation was carried out according to a previous report^30^. Briefly, 200 μg of the sample was dissolved in 5 mL of loading buffer (ACN/H_2_O/TFA = 89:10.5:0.5, v/v/v), incubated with 20 mg of Fe_3_O_4_-GO@PDA-Chitosan nanocomposites for 1 h, and washed thrice with 2 mL of loading buffer. Then, the trapped glycopeptides were eluted twice with 400 μL of elution buffer, and the elution was evaporated to dryness. The obtained glycopeptides were redissolved in 10 mM of NH_4_HCO_3_, and glycan moieties were removed by 1000 unites of PNGase F. The mixture was desalted and enriched using Sep-pak C18 cartridges (Waters Ltd., Elstree, UK), evaporated to dryness, and redissolved prior to anlysis by nano LC-MS/MS.

## Results and Discussion

### Preparation and characterization of Fe_3_O_4_-GO@PDA-Chitosan nanocomposites

**Figure 2:**
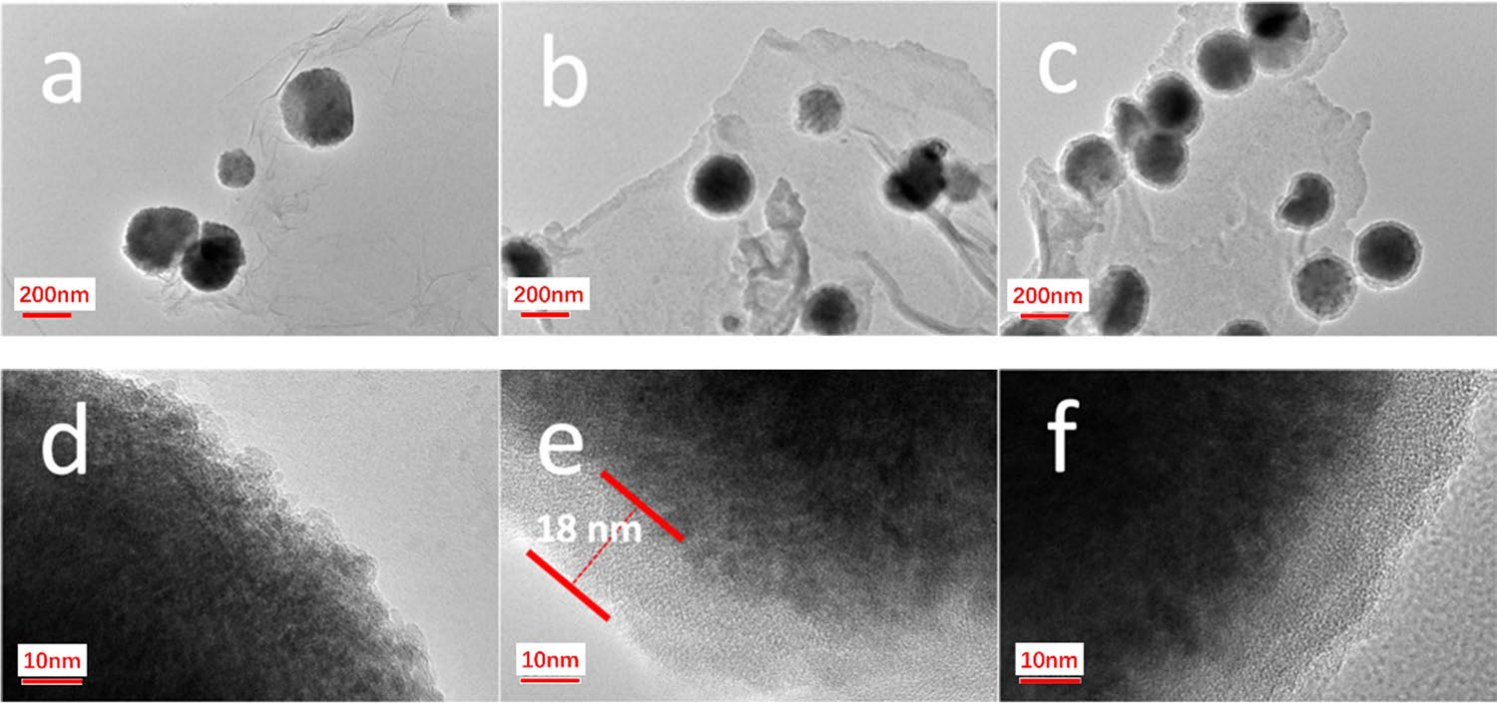
TEM images of Fe_3_O_4_-GO (**a,d**), Fe_3_O_4_-GO@PDA (**b,e**) and Fe_3_O_4_-GO@PDA-Chitosan (**c,f**) nanocomposites.

The morphology of Fe_3_O_4_-GO, Fe_3_O_4_-GO@PDA, Fe_3_O_4_-GO@PDA-Chitosan nanocomposites and the thickness of the grafted PDA layer were revealed by transmission electron microscopy (TEM) at the 200 or 10 nanometer scale. As shown in Fig. 2a,d, Fe_3_O_4_ nanoparticles were irregularly interspersed on the graphene nanosheets, indicating the successful assembly of Fe_3_O_4_-GO nanocomposites. By means of self-polymerization, the PDA layer was coated on the surface of Fe_3_O_4_-GO with the thickness of ~18 nm (Fig. 2b,e). The molecular weight of degraded chitosan was determined to be ~1471 g·mol^−1^ by Gel Permeation Chromatography, so the modification of degraded chitosan on the surface of Fe_3_O_4_-GO@PDA nanocomposites was not detected. When the chitosan shell was formed on the surface of Fe_3_O_4_-GO@PDA, the obtained Fe_3_O_4_-GO@PDA-Chitosan displayed an obvious core-shell structure (Fig. 2c,f).

**Figure 3:**
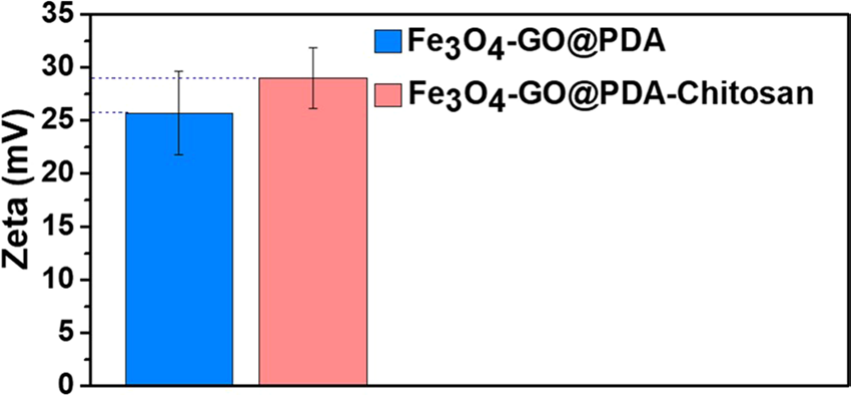
Zeta potential of Fe_3_O_4_-GO@PDA and Fe_3_O_4_-GO@PDA-Chitosan nanocomposites.

The Zeta potentials of the obtained nanocomposites were detected in acid aqueous solution (H_2_O/TFA = 99.5:0.5, v/v). The zeta potential of Fe_3_O_4_-GO@PDA was 25.70 mV (Fig. 3), which indicated that there were abundant phenolic group and amino groups onto the surface of PDA layer. And after graft of chitosan on the surface of PDA layer, the zeta potential increased to 29.01 mV due to the addition of amine and hydroxyl groups.

The magnetic properties of the obtained nanocomposites were measured by a vibrating sample magnetometer (VSM) at room temperature. The saturation magnetic (*M*_*s*_) values of Fe_3_O_4_-GO, Fe_3_O_4_-GO@PDA and Fe_3_O_4_-GO@PDA-Chitosan nanocomposites were 34.06, 19.66, 16.21 emu·g^−1^, respectively (Fig. S1, Electronical Supporting Materials). Although the saturation magnetization value of Fe_3_O_4_-GO@PDA-Chitosan was reduced to 16.21 emu·g^−1^, the Fe_3_O_4_-GO@PDA-Chitosan nanocomposites can be quickly separated within 10 s under an external magnetic field (Fig. S1 inset, Electronical Supporting Materials) and re-dispersed quickly after removal of the magnetic field.

The crystalline phases of Fe_3_O_4_-GO, Fe_3_O_4_-GO@PDA and Fe_3_O_4_-GO@PDA-Chitosan nanocomposites were characterized by the XRD technique (Fig. S2, Electronical Supporting Materials). Six discernible diffraction peaks at 30.06°, 35.36°, 43.16°, 53.42°, 57.08°, 62.50° were index as (220), (311), (400), (422), (511) and (440) in the database of the Joint Committee on Powder Diffraction Standards (JCPDS card: 19-0629). This result indicated that the presence of Fe_3_O_4_ nanoparticles in the nanocomposites.

**Figure 4:**
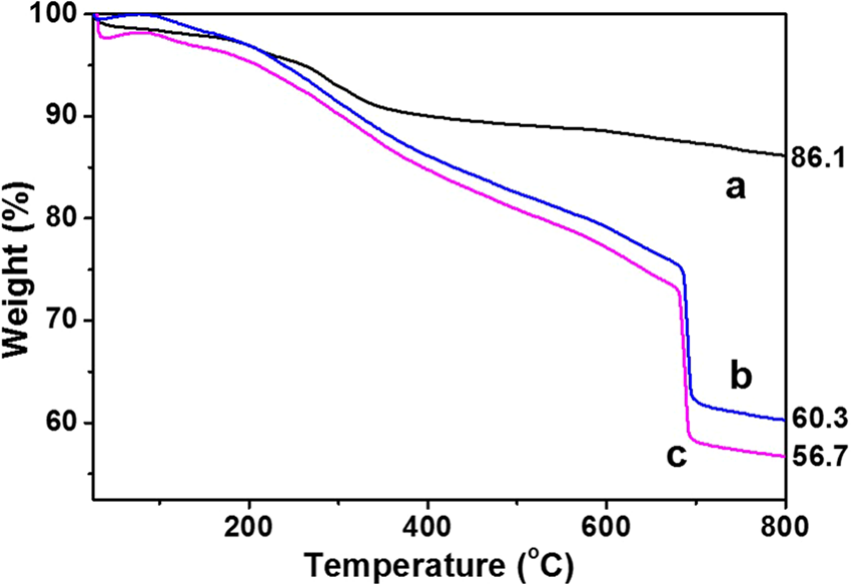
TGA curves of Fe_3_O_4_ (**a**), Fe_3_O_4_-GO@PDA (**b**) and Fe_3_O_4_-GO@PDA-Chitosan (**c**) nanocomposites.

The thermogravimetric analysis (TGA) curves of Fe_3_O_4_-GO, Fe_3_O_4_-GO@PDA and Fe_3_O_4_-GO@PDA-Chitosan nanocomposites are shown in Fig. 4. It can be found that 13.9% weight loss occurred for Fe_3_O_4_-GO (Fig. 4a) corresponding to the content of GO. And there were 39.7 and 43.3% weight loss for Fe_3_O_4_-GO@PDA (Fig. 4b) and Fe_3_O_4_-GO@PDA-Chitosan (Fig. 4c), respectively. From the data of TGA curves, the amount of immobilized chitosan onto the Fe_3_O_4_-GO@PDA-Chitosan nanocomposites was calculated to 24.5 μmol·g^−1^.

**Figure 5:**
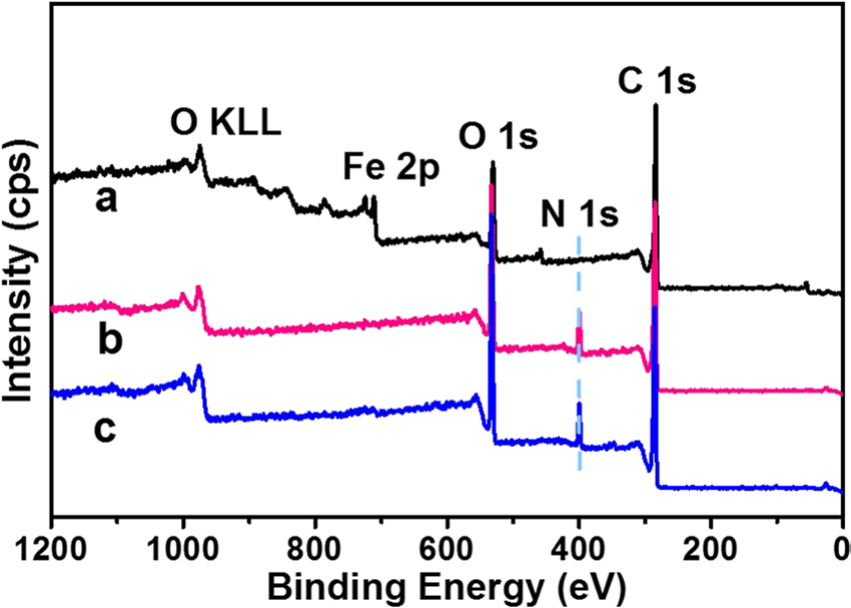
XPS spectrum of Fe_3_O_4_ (**a**), Fe_3_O_4_-GO@PDA (**b**) and Fe_3_O_4_-GO@PDA-Chitosan (**c**) nanocomposites.

**Figure 6:**
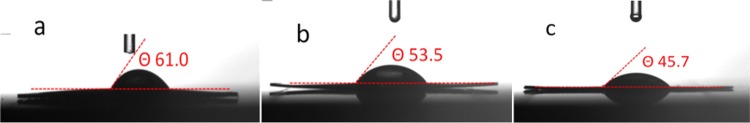
Water contact angles of Fe_3_O_4_ (**a**), Fe_3_O_4_-GO@PDA (**b**) and Fe_3_O_4_-GO@PDA-Chitosan (**c**) nanocomposites.

The surface composition of the obtained nanocomposites (Fe_3_O_4_-GO, Fe_3_O_4_-GO@PDA, Fe_3_O_4_-GO@PDA-Chitosan) were characterized by XPS. As shown in Fig. 5, the peaks of Fe 2p, O 1 s, N 1 s and C 1 s were evidently observed. The contact angle of prepared nanocomposites (Fe_3_O_4_-GO, Fe_3_O_4_-GO@PDA, Fe_3_O_4_-GO@PDA-Chitosan) were measured with the powder tableting method (Fig. 6) to evaluate the relative surface hydrophilicity. The contact angle of Fe_3_O_4_-GO (a), Fe_3_O_4_-GO@PDA (b) and Fe_3_O_4_-GO@PDA-Chitosan (c) nanocomposites were 61.0°, 53.5°, and 45.7°, respectively. It indicated that the polydopamine layer coated on the surface of magnetic graphene played a role not only in facile synthesis procedure via Michael addition, but also in hydrophilicity raise; the modification of chitosan improved further the hydrophilicity of the nanocomposites.

### Glycopeptide enrichment from standard proteins by Fe_3_O_4_-GO@PDA-Chitosan nanocomposites

**Figure 7:**
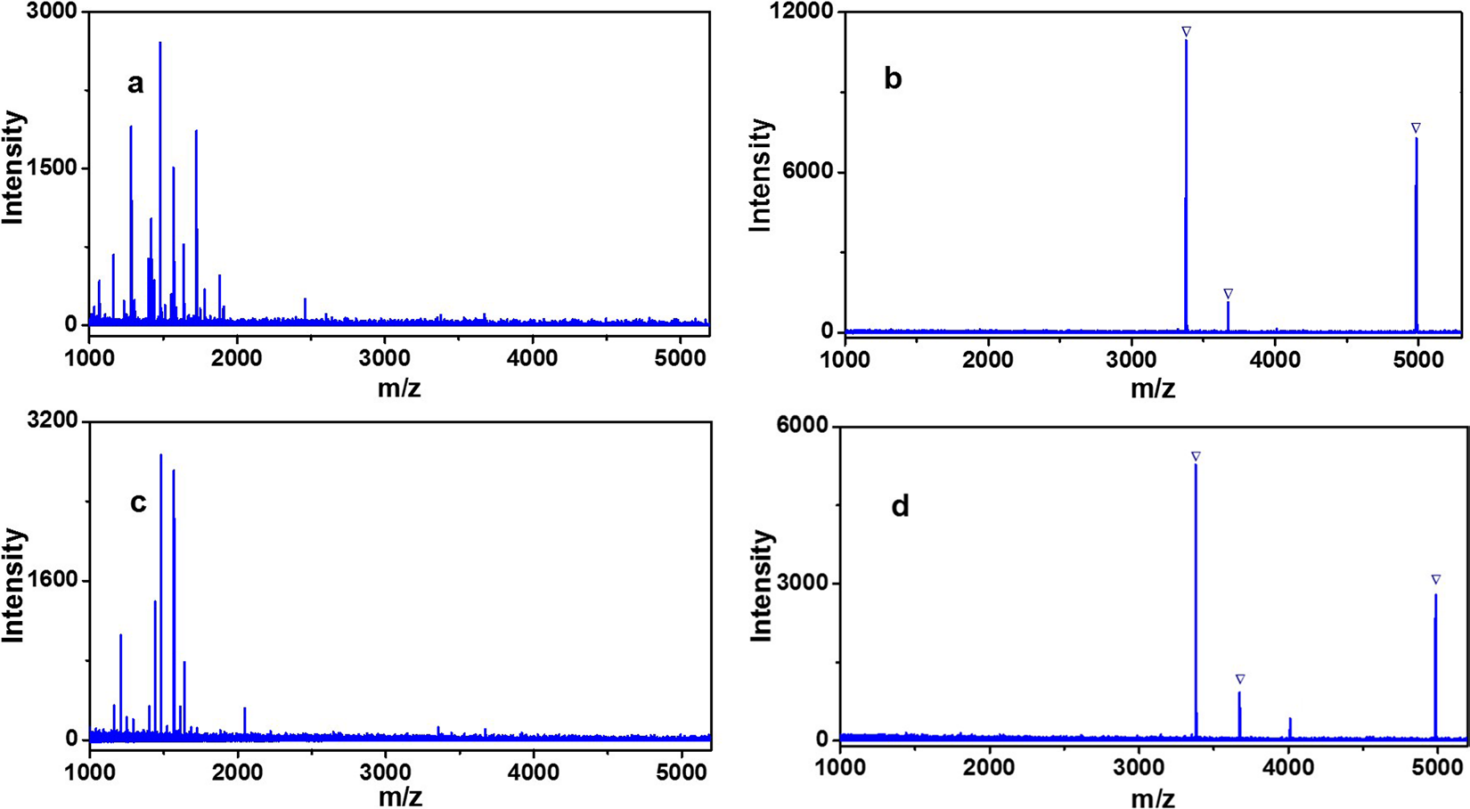
MALDI-TOF mass analysis of tryptic digest of HRP (50 fmol·μL^−1^): (**a**) before enrichment, (**b**) eluent and (**c**) supernatant after enrichment with Fe_3_O_4_-GO@PDA-Chitosan nanocomposites; eluent after enrichment with (**d**) Fe_3_O_4_-GO@PDA nanocomposites. The peaks of glycopeptides are marked with .

To select the optimal enrichment condition to glycopeptides, three different kinds of loading buffer (89% ACN/H_2_O, 0.1% TFA; 89% ACN/H_2_O, 0.5% TFA; 89% ACN/H_2_O, 1% TFA) were utilized for investigate the effect of capturing glycopeptides from HRP digestion by Fe_3_O_4_-GO@PDA-Chitosan nanocomposites and the results were displayed in Fig. S3 (Electronical Supporting Materials). The performance of Fe_3_O_4_-GO@PDA-Chitosan nanocomposites for enrichment of N-glycopeptides was the best in the loading buffer (89% ACN/H_2_O, 0.5% TFA). By taking advantage of the optimized enrichment condition, Fe_3_O_4_-GO@PDA-Chitosan nanocomposites were applied to capture N-glycopeptides from standard HRP tryptic digest (50 fmol·μL^−1^). As shown in Fig. 7a, the direct analysis of HRP digest (50 fmol·μL^−1^) without enrichment step, the signal peaks of low abundance of N-glycopeptides were completely suppressed, no target analyte was detected. After enrichment by Fe_3_O_4_-GO@PDA-Chitosan nanocomposites, the number and signal intensity of glycopeptides were distinctly enhanced, meanwhile, three N-glycopeptides were detected and non-glycopeptides were completely removed (Fig. 7b, detailed information is listed in Table S1, Electronical Supporting Materials). And no glycopeptides peaks were found in the supernatant (Fig. 7c). For comparison, three N-glycopeptides were also identified after enrichment with Fe_3_O_4_-GO@PDA nanocomposites, but the signal intensity was weaker and the one signal peak of non-glycopeptide appeared in MS spectra (Fig. 7d).

**Figure 8:**
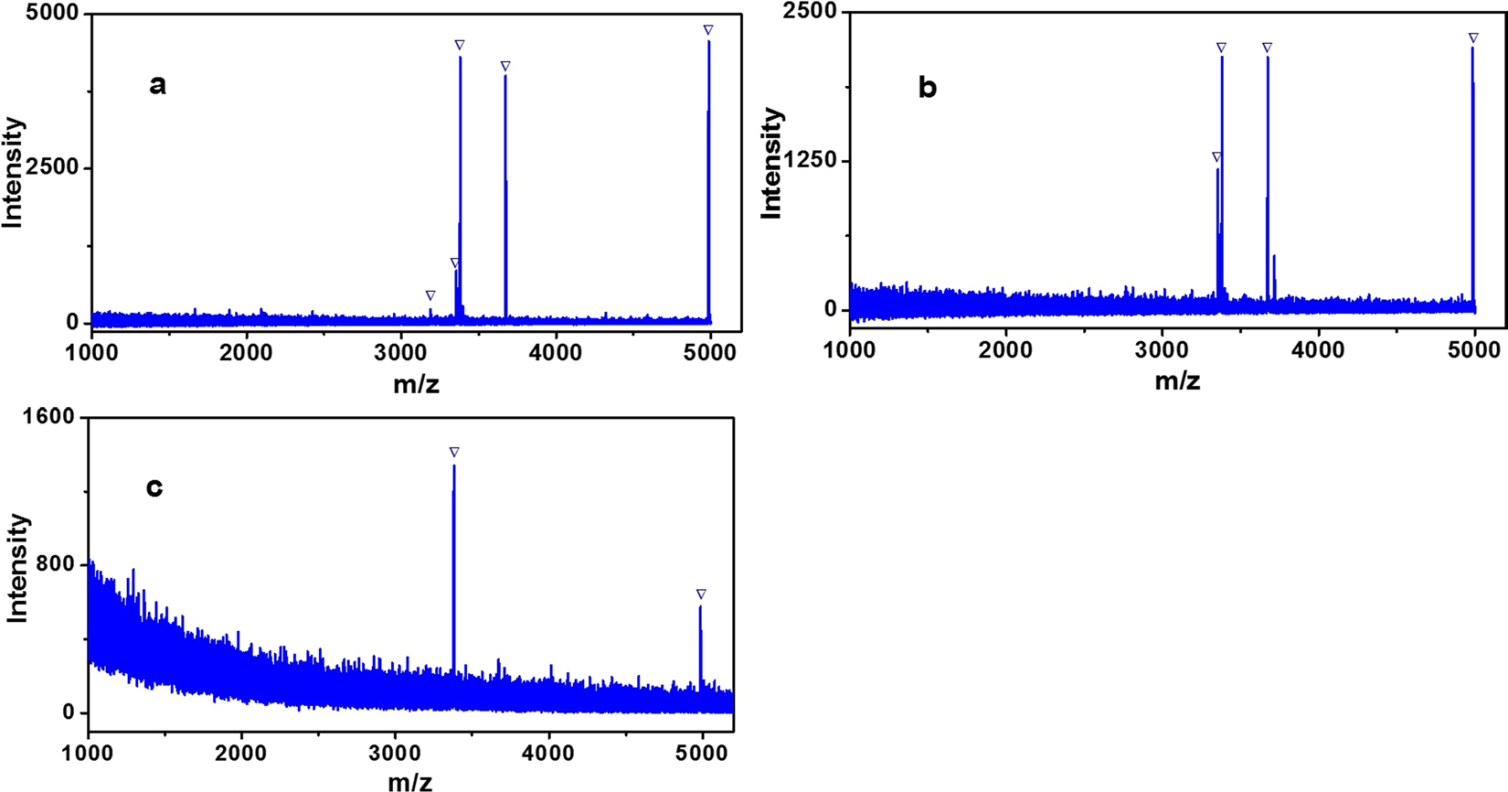
MALDI-TOF mass analysis of tryptic digest of HRP (0.5 fmol·μL^−1^) after enrichment with Fe_3_O_4_-GO@PDA-Chitosan (**a**) and Fe_3_O_4_-GO@PDA nanocomposites (**b**); MALDI-TOF mass analysis of tryptic digest of HRP (0.4 fmol·μL^−1^) after enrichment with Fe_3_O_4_-GO@PDA-Chitosan(**c**) nanocomposites. The peaks of glycopeptides are marked with .

The detection sensitivity of Fe_3_O_4_-GO@PDA-Chitosan (or Fe_3_O_4_-GO@PDA) nanocomposites for N-glycopeptides enrichment was evaluated with lower concentrations of HRP tryptic digests. When the concentration of HRP digestion decreased to 0.5 fmol·μL^−1^, five N-glycopeptides were detected after enrichment with Fe_3_O_4_-GO@PDA-Chitosan nanocomposites, and four N-glycopeptides and one non-glycopeptide were detected after enrichment with Fe_3_O_4_-GO@PDA nanocomposites (Fig. 8a,b). Even at the concentration of 0.4 fmol·μL^−1^, two N-glycopeptides were still observed after treatment of Fe_3_O_4_-GO@PDA-Chitosan nanocomposites (Fig. 8c). The detection sensitivity of Fe_3_O_4_-GO@PDA-Chitosan nanocomposites was higher than some reported hydrophilic nanomaterials, such as Au NP-maltose/PDA/Fe_3_O_4_-RGO (2.5 fmol·μL^−1^)^29^, Fe_3_O_4_-DA-Maltose (1.25 fmol·μL^−1^)^31^, Fe_3_O_4_@mSiO_2_@G6P (0.5 fmol·μL^−1^)^32^, mMOF@Au@GSH (0.5 fmol·μL^−1^)^20^.

**Figure 9:**
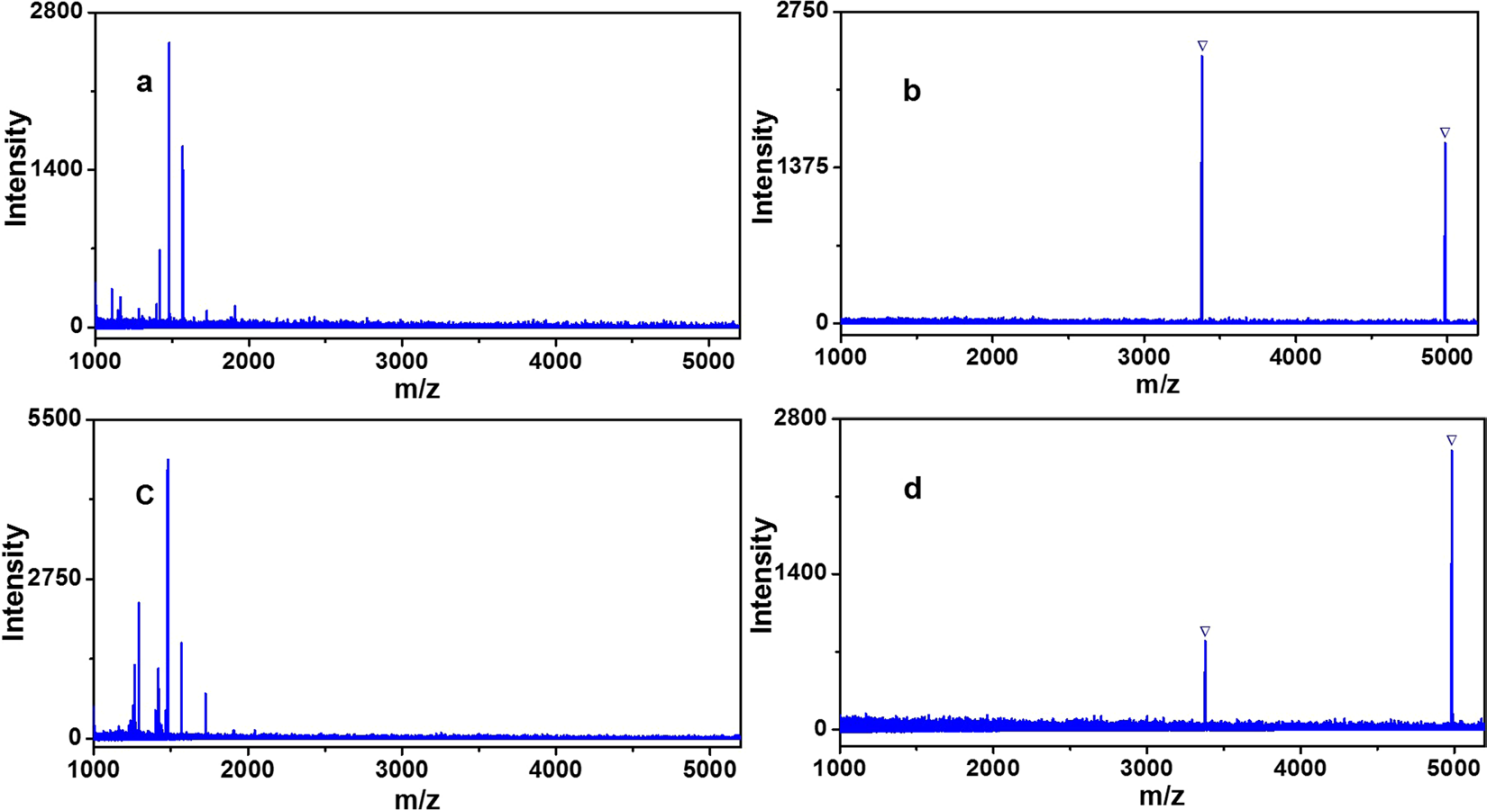
MALDI-TOF mass spectra of mixture of tryptic HRP and BSA before enrichment: (**a**) 1:1; (**c**) 1:10. MALDI-TOF MS spectra of the identified glycopeptides enriched by Fe_3_O_4_-GO@PDA-Chitosan nanocomposites from the tryptic mixture of HRP and BSA with the molar ratio of (**b**) 1:1; (**d**) 1:10. The peaks of glycopeptides are marked with .

The enrichment selectivity of Fe_3_O_4_-GO@PDA-Chitosan nanocomposites was further investigated, by evaluating the capture performance for N-glycopeptides from the mixture of HRP and BSA tryptic digest with different molar ratios. When the molar ratio of digests mixture of HRP and BSA was 1:1 or 1:10, the MS signals of N-glycopeptides were completely suppressed in direct analysis due to the strong signal suppression from an amount of non-glycopeptides (Fig. 9a,c). By contrast, after enrichment by Fe_3_O_4_-GO@PDA-Chitosan nanocomposites, two N-glycopeptides derived from HRP were detected with no non-glycopeptide signals (Fig. 9b). The molar ratio of HRP and BSA was further increased even 1:10, an overwhelming majority of non-glycopeptides were removed and the peaks of N-glycopeptides still absolutely dominated MS spectra (Fig. 9d). The enrichment selectivity of Fe_3_O_4_-GO@PDA-Chitosan nanocomposites was higher than some reported hydrophilic nanomaterials functionalized with saccharides, such as Au NP-maltose/PDA/Fe_3_O_4_-RGO^29^, Fe_3_O_4_-DA-Maltose^31^, MagG/Au/Glu^33^. The results indicated that Fe_3_O_4_-GO@PDA-Chitosan nanocomposites have the great potential in N-glycopeptides enrichment from complex biological sample.

**Figure 10:**
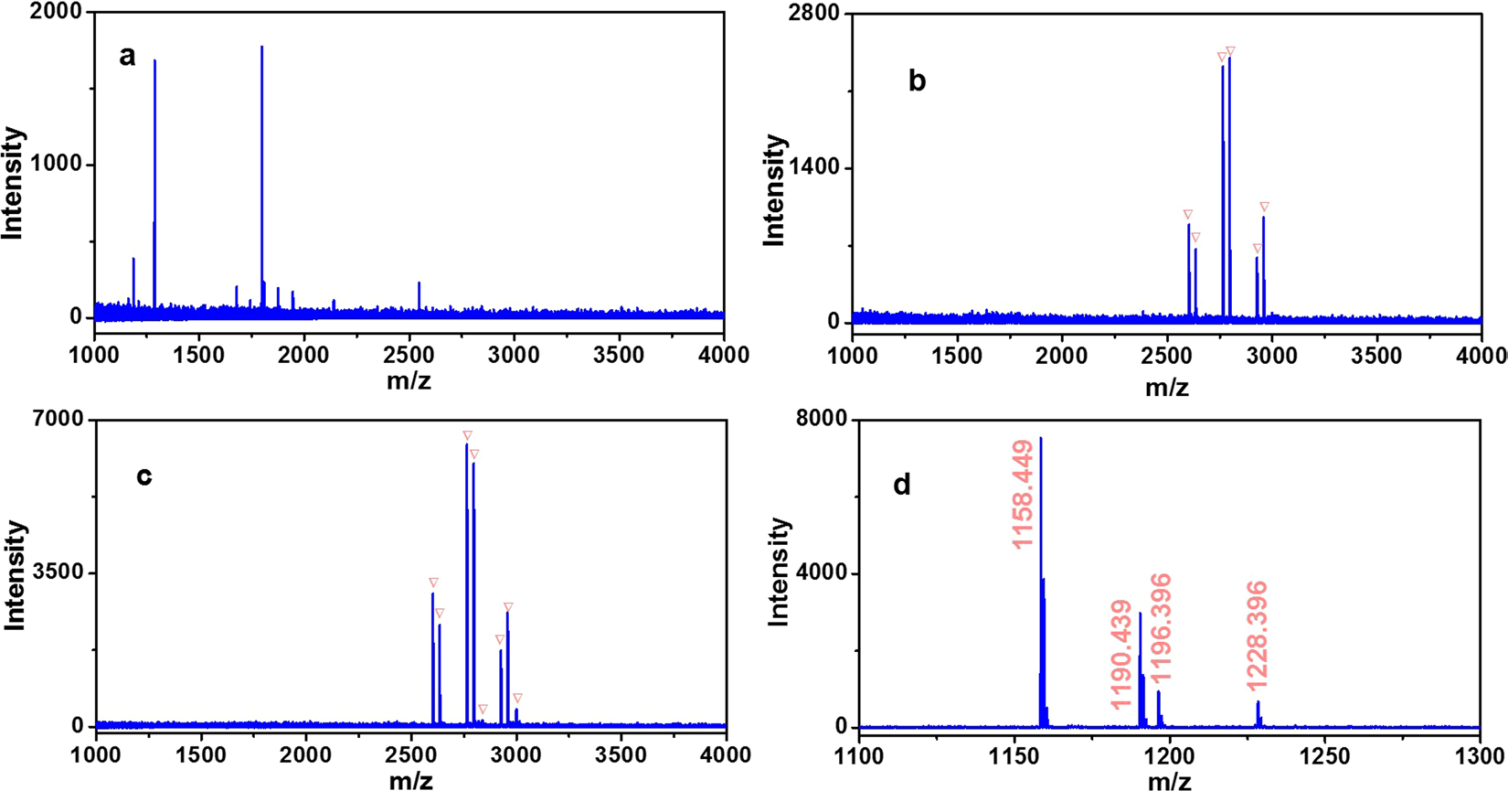
MALDI-TOF mass spectra of tryptic digests of IgG (5 fmol·μL^−1^): direct analysis (**a**); analysis after enrichment with Fe_3_O_4_-GO@PDA (**b**) or Fe_3_O_4_-GO@PDA-Chitosan nanocomposites (**c**), and deglycosylation by PNGase F (**d**). The peaks of glycopeptides are marked with .

To demonstrate the unbiased enrichment performance of Fe_3_O_4_-GO@PDA-Chitosan nanocomposites towards different N-glycan, it was applied to capture N-glycopeptides from IgG digest, which contains different glycoforms from HRP. In direct MS analysis without enrichment, no N-glycopeptides were detected on account of the strong interference from non-glycopeptides (Fig. 10a). Seven N-glycopeptides were identified after enrichment with Fe_3_O_4_-GO@PDA nanocomposites (Fig. 10b). For comparison, fifteen N-glycopeptides were detected after enrichment by Fe_3_O_4_-GO@PDA-Chitosan nanocomposites (Fig. 10c, detailed information is listed in Table S2, Electronical Supporting Materials), and the signal intensity was stronger in MS spectra. The eluted glycopeptides from Fe_3_O_4_-GO@PDA-Chitosan nanocomposites enrichment were deglycosylated by PNGase F and two strong signals of deamidated peptides (at m/z 1158.4 (EEQFN#STFR), 1190.4 (EEQYN#STFR)) were detected (Fig. 10d), which demonstrated that the enriched peptide fragments were N-glycopeptides.

### Evaluation of binding capacity of Fe_3_O_4_-GO@PDA-Chitosan nanocomposites for N-glycopeptide enrichment

**Figure 11:**
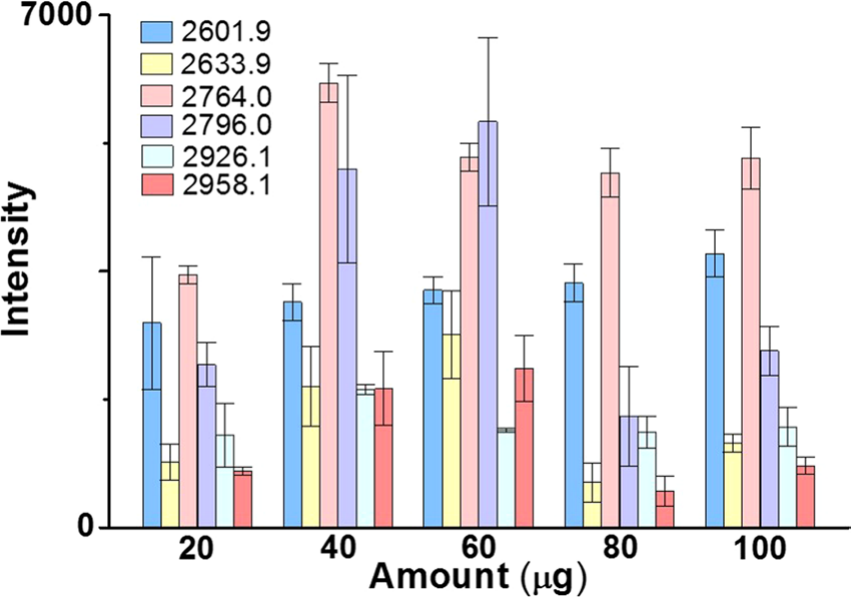
The intensity of six selected N-glycopeptides from tryptic digests of human IgG (3 μg) after enrichment by different amount of Fe_3_O_4_-GO@PDA-Chitosan nanocomposites.

Different masses (20-100 μg) of Fe_3_O_4_-GO@PDA-Chitosan nanocomposites were dispersed with ultrasonication for 6 s in the loading buffer (400 μL, V(ACN)/V(H_2_O)/V(TFA) = 89:10.5:0.5) containing human IgG digest (3 μg). After the enrichment, the elution was analyzed with MALDI-TOF MS. When the MS signal intensity of six selected glycopeptides reach maximum, the total amount of glycopeptides were captured by the nanocomposites. The binding capacity was calculated by the mass ratio of human IgG (3 μg) and the Fe_3_O_4_-GO@PDA-Chitosan nanocomposites. As shown in Fig. 11, the N-glycopeptides from human IgG digest (3 μg) were almost bonded onto 40 μg of Fe_3_O_4_-GO@PDA-Chitosan nanocomposites, and the binding capacity of the nanocomposites was about 75 mg·g^−1^, Which was higher than some reported hydrophilic materials modified with saccharides such as Fe_3_O_4_@CS MCNCs (17.5 mg·g^−1^)^25^ and Fe_3_O_4_-DA-Maltose (43 mg·g^−1^)^31^. Meanwhile, the binding capacity of Fe_3_O_4_-GO@PDA nanocomposites was evaluated, the N-glycopeptides from human IgG digest (3 μg) were almost bonded onto 80 μg of Fe_3_O_4_-GO@PDA nanocomposites, and the binding capacity of the nanocomposites was about 37.5 mg·g^−1^ (Fig. S4, Electronical Supporting Materials).

### The stability and reusability of Fe_3_O_4_-GO@PDA-Chitosan nanocomposites

To evaluate the stability and reusability of Fe_3_O_4_-GO@PDA-Chitosan nanocomposites, the material was firstly placed in a glass vial for three months at room temperature, and then applied to capture N-glycopeptides from HRP digest for four cycles. As shown in Fig. S5 (Electronical Supporting Materials), the enrichment performance of Fe_3_O_4_-GO@PDA-Chitosan nanocomposites in the first time and fourth time was all good, which indicates that the Fe_3_O_4_-GO@PDA-Chitosan nanocomposites own good stability and repeatability.

### Practical application of Fe_3_O_4_-GO@PDA-Chitosan nanocomposites on glycopeptide enrichment

In order to test the practical application of Fe_3_O_4_-GO@PDA-Chitosan nanocomposites on glycopeptide enrichment in the complex samples, the Fe_3_O_4_-GO@PDA-Chitosan nanocomposites were applied to analyze the N-linked glycopeptides from the human renal mesangial cells (HRMC). HRMC serve as a filtration barrier of the kidney. The injury of mesangial cells could cause diabetic nephropathy, leading end-stage renal disease. Emerging evidence indicates that mesangial cells can be damaged by high glucose, however the mechanism is unclear. In this work, Fe_3_O_4_-GO@PDA-Chitosan nanocomposites were used to enrich the N-link glycopeptide from the HRMC which have been treated by high glucose and the glycopeptide enrichment was further identified by HPLC-MS/MS (The details of cell culture, protein extraction and MS/MS data analysis in the Electronical Supporting Materials). Finally, 393 N-linked glycopeptides, representing 195 different glycoproteins and 458 glycosylation sites were identified in HRMC (Table S3, Electronical Supporting Materials).

## Conclusion

In summary, the novel chitosan-interspersed magnetic nanocomposites (Fe_3_O_4_-GO@PDA-Chitosan) were prepared successfully via a facile two-step method for selective enrichment of N-glycopeptides. Due to the enhanced hydrophilicity by the PDA layer and chitosan, the resulting nanocomposites exhibited good selectivity and sensitivity for N-glycopeptides enrichment from the complex biological samples. The combination of dopamine self-polymerization and Michael addition is a feasible strategy to assemble hydrophilic nanomaterials applied in N-glycopeptides enrichment.

## Supplementary information

Supplementary Information.
