# Supplementary material for: Facile synthesis of hydrophilic magnetic graphene nanocomposites via dopamine self-polymerization and Michael addition for selective enrichment of N-linked glycopeptides
Source: Sci Rep. 2020 Jan 9;10:71. doi: 10.1038/s41598-019-56944-4 (PMC6952460; doi:10.1038/s41598-019-56944-4)
Supplement: Supplementary file 1 — Supplementary Information. [file 41598_2019_56944_MOESM1_ESM.docx]

**Electronical Supporting Materials**

**Facile synthesis of hydrophilic magnetic graphene nanocomposites via dopamine self-polymerization and Michael addition for selective enrichment of N-linked glycopeptides**

Changfen Bi^1^, Ye Yuan^1^, Yuran Tu^2^, Jiahui Wu^2^, Yulu Liang^2^, Yiliang Li^*1^, Xiwen He^2^, Langxing Chen^*2,3^, and Yukui Zhang^4^

^1^Tianjin Key Laboratory of Radiation Medicine and Molecular Nuclear Medicine, Institute of Radiation Medicine, Peking Union Medical College & Chinese Academy of Medical Sciences, Tianjin 300192, China

^2^College of Chemistry, Tianjin Key Laboratory of Biosensing and Molecular Recognition, State Key Laboratory of Medicinal Chemical Biology, Nankai University, Tianjin 300071, China

Fax: (+86) 22-2350-2458, E-mail: [lxchen@nankai.edu.cn](mailto:lxchen@nankai.edu.cn)

^3^Collaborative Innovation Center of Chemical Science and Engineering (Tianjin), Tianjin 300071, China

^4^Dalian Institute of Chemical Physics, Chinese Academy of Sciences, Dalian 116023, China

1. **Cell culture and protein extraction**

Human renal mesangial cells (HRMC, kindly donated by Dr. Mingzhen Li (Metabolic Diseases Hospital, Tianjin Medical University, Tianjin, China)) were grown in Dulbeco’s modified eagle medium (Thermo Fisher) supplemented with 1 % penicillin/streptomycin and 10 % fetal bovine serum in a humidified atmosphere with 5 % CO_2_ in air. After collection, HRMC were washed twice with cold phosphate-buffer saline (PBS). The cell layers were further solubilized in RIPA buffer (0.1 SDS, 1 % Triton X-100 and 1 % deoxycholate containing proteinase inhibitors (sodium butyrate and 1 mmol/L phenylmethylsulfonyl fluoride (PMSF, Sigma-Aldrich)). Then total cell undissolved substance was ultrasound. The crude extract was finally clarified by centrifugation at 16,000×g at 4 °C for 10 min. The concentration of total proteins was determined by BCA protein assay kit (Pierce BCA protein assay kit, Thermo Scientific).

1. **LC-MS/MS analysis**

The deglycosylated peptides enriched from HRMC tryptic digest was desalted using a μ-C18 Ziptip and dissolved in 10 μL of HPLC buffer A (0.1 % (v/v) formic acid in water). 5 μL sample was injected into a Nano-LC system (EASY-nLC 1000, Thermo Fisher Scientific, Waltham, MA). Chromatography was performed using an EASY -Spray Nano-LC source with a 15 cm × 50 μm inner diameter column packed with 2 μm C18 particles. The flow rate was 300 nl/min, a 45-min linear gradient from 2 to 35 % HPLC buffer B (0.1 % formic acid in CH_3_CN) was developed, eventually the organic content was increased to 50 % over 10 min. The HPLC elute was electrosprayed directly into an Orbitrap Q-Exactive mass spectrometer (Thermo Fisher Scientific, Waltham, MA). The source was operated at 1.8 kV. For full MS survey scan, automatic gain control (AGC) target was 3e6, scan range was from 350 to 1750 with the resolution of 70,000. The 10 most intense peaks with charge state 2 and above were selected for fragmentation by high-energy collision dissociation (HCD) with normalized collision energy of 27 %. The MS2 spectra were acquired with 17.500 resolution.

1. **MS/MS Data analysis**

Raw file was searched against the Uniport-Human protein sequence database using the PD search engine (version 2.1.0, Thermo Fisher Scientific) with an overall false discovery rate (FDR) for peptide of less than 1 %. Trypsin was specified as digesting enzyme. A maximum of 2 missing cleavages were allowed. Mass tolerance for precursor ions were set at ±10 ppm for precursor ions and ±0.02 Da for MS/MS. Oxidation of methionine, acetylation on protein N-terminal, and asparagines deamination were fixed as variable modifications. Carbamidomethylation on Cys was specified as fixed modification.


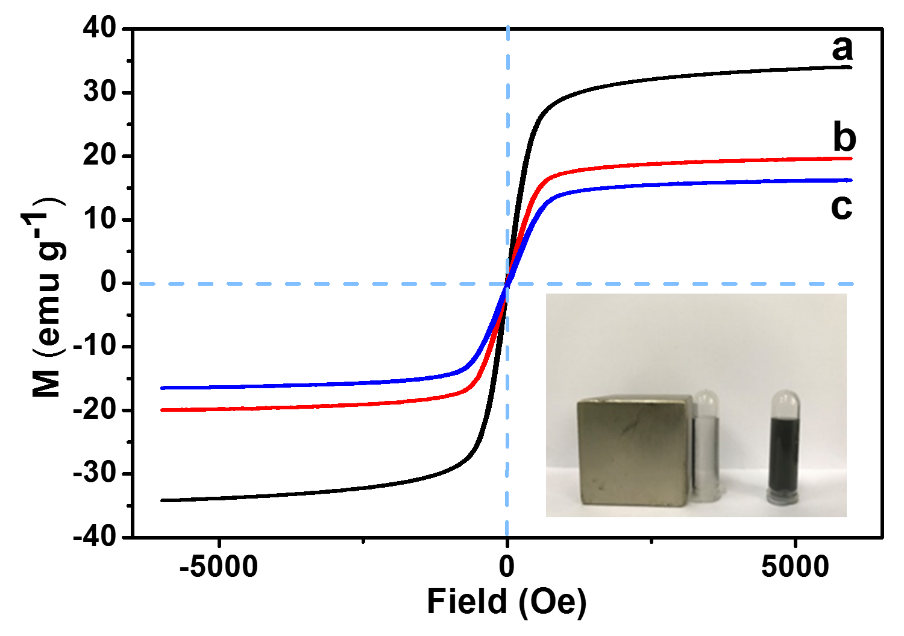


**Figure S1** Magnetic hysteresis curves of Fe_3_O_4_-GO (a), Fe_3_O_4_-GO@PDA (b) and Fe_3_O_4_-GO@PDA-Chitosan (c) nanocomposites.

**Figure S2** XRD patterns of Fe_3_O_4_-GO (a), Fe_3_O_4_-GO@PDA (b) and Fe_3_O_4_-GO@PDA-Chitosan (c) nanocomposites.


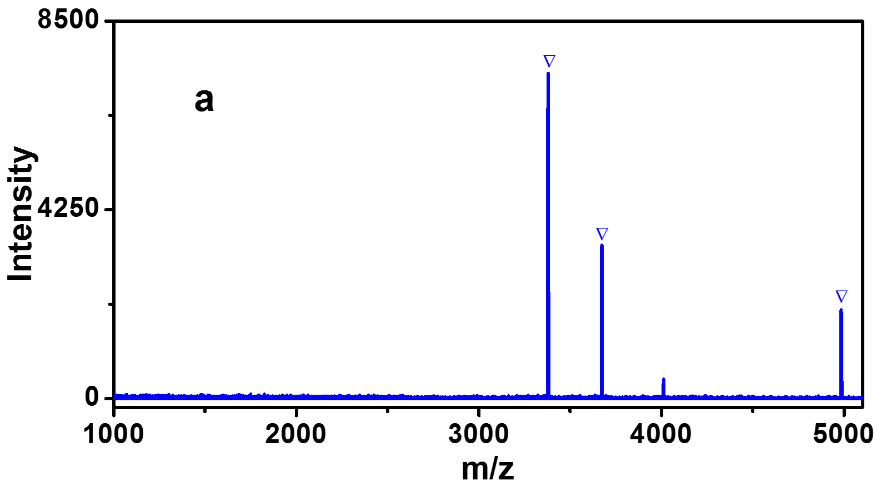

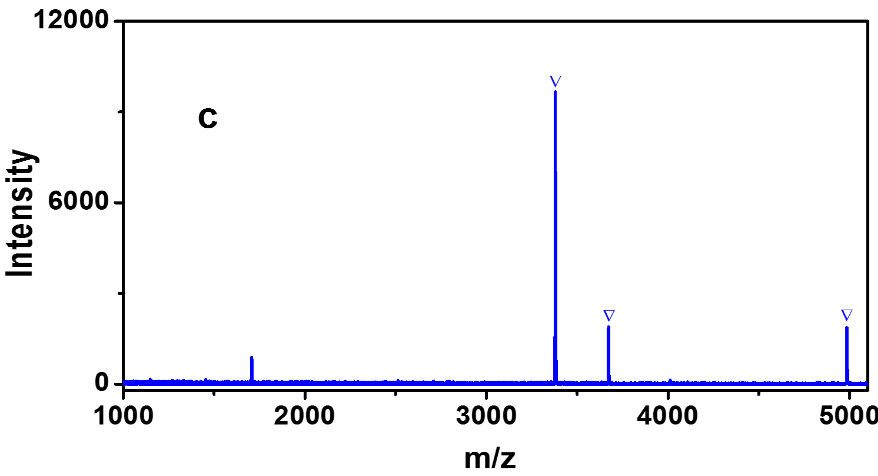


**Figure S3** MALDI-TOF mass analysis of the N-glycopeptides derived from HRP tryptic digests after treatment with Fe_3_O_4_-GO@PDA-Chitosan nanocomposites with different loading buffer, (a) 89% ACN/H_2_O, 0.1% TFA; (b) 89% ACN/H_2_O, 0.5% TFA; (c) 89% ACN/H_2_O, 1% TFA. N-glycopeptides were marked with
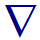
.

**Figure S4** The intensity of six selected N-glycopeptides from tryptic digests of human IgG (3 μg) after enrichment by different amount of Fe_3_O_4_-GO@PDA nanocomposites.

**Figure S5** MALDI-TOF mass analysis of the glycopeptides derived from HRP tryptic digest: (a) after treatment with Fe_3_O_4_-GO@PDA-Chitosan nanocomposites used for the first time, (b) after treatment with Fe_3_O_4_-GO@PDA-Chitosan nanocomposites used for the fourth time. The peaks of glycopeptides are marked with
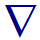
.

**Table S1** Observed N-glycopeptides and glycan structures of human HRP digests enriched by Fe_3_O_4_-GO@PDA-Chitosan nanocomposites. The N-glycosylation sites are marked with N#.

| Peak number | Observed  m/z | Glycan composition | Amino acid sequence |
| --- | --- | --- | --- |
| 1 | 3189.2 | [Hex]2[HexNAc]2[Fuc]1[Xyl]1 | SFAN#STQTFFNAFVEAMDR |
| 2 | 3355.1 | [Hex]2[HexNAc]2[Fuc]1[Xyl]1 | SFAN#STQTFFNAFVEAMDR |
| 3 | 3370.2 | [Hex]3[HexNAc]2[Fuc]1[Xyl]1 | SFAN#STQTFFNAFVEAMDR |
| 4 | 3380.4 | [Hex]2[HexNAc]2[Fuc]1 | GLIQSDQELFSSPN#ATDTIPLVR |
| 5 | 3672.4 | [Hex]3[HexNAc]2[Fuc]1[Xyl]1 | GLIQSDQELFSSPN#ATDTIPLVR |
| 6 | 4986.1 | [Hex]3[HexNAc]2[Fuc]1[Xyl]1  [Hex]3[HexNAc]2[Fuc]1[Xyl]1 | LYN#FSNTGLPDPTLN#TTYLQTLR |

Hex = mannose, HexNAc = N-acetylglucosamine, Fuc = fucose, Xyl = xylose.

**Table S2** Observed N-glycopeptides and glycan structures of human IgG digests enriched by Fe_3_O_4_-GO@PDA-Chitosan nanocomposites. The N-glycosylation sites are marked with N#.

| Peak  number | Observed  m/z | Glycan composition | Amino acid sequence |
| --- | --- | --- | --- |
| 1 | 2601.9 | [Hex]3[HexNAc]4[Fuc]1 | EEQFN#STFR |
| 2 | 2633.9 | [Hex]3[HexNAc]4[Fuc]1 | EEQYN#STYR |
| 3 | 2650.7 | [Hex]4[HexNAc]4 | EEQYN#STYR |
| 4 | 2763.9 | [Hex]4[HexNAc]4[Fuc]1 | EEQFN#STFR |
| 5 | 2795.9 | [Hex]4[HexNAc]4[Fuc]1 | EEQYN#STYR |
| 6 | 2805.8 | [Hex]3[HexNAc]5[Fuc]1 | EEQYN#STFR |
| 7 | 2812.0 | [Hex]5[HexNAc]4 | EEQYN#STYR |
| 8 | 2819.8 | [Hex]4[HexNAc]5 | EEQFN#STFR |
| 9 | 2838.7 | [Hex]3[HexNAc]5[Fuc]1 | EEQYN#STYR |
| 10 | 2926.1 | [Hex]5[HexNAc]4[Fuc]1 | EEQFN#STFR |
| 11 | 2957.9 | [Hex]5[HexNAc]4[Fuc]1 | EEQYN#STYR |
| 12 | 2968.4 | [Hex]4[HexNAc]5[Fuc]1 | EEQFN#STFR |
| 13 | 2982.9 | [Hex]5[HexNAc]5 | EEQFN#STFR |
| 14 | 2999.0 | [Hex]4[HexNAc]5[Fuc]1 | EEQYN#STYR |
| 15 | 3014.8 | [Hex]5[HexNAc]5 | EEQYN#STYR |

Hex= mannose, HexNAc= N-acetylhexosamine, Fuc= fucose, NeuAc= N-acetylneuraminic acid.

**Table S3** List of identified glycoproteins and peptides sequence from tryptic digest of proteins sample extracted from human renal mesangial cells after enrichment by Fe_3_O_4_-GO@PDA-Chitosan nanocomposites.

N# denotes the N-linked glycosylation site.

| No | Protein Group Accessions | Peptide Sequence |
| --- | --- | --- |
| 1 | P62258 | YLAEFATGN#DRKEAAEN#SLVAYK |
| 2 | P08123 | KAVILQGSN#DVELVAEGNSR |
| 3 | P22314 | GLGVEIAKN#IILGGVK |
| 4 | P07355 | GVDEVTIVN#ILTNR |
| 5 | P78371 | MLPTIIADN#AGYDSADLVAQLR |
| 6 | P18206 | AAAVGTAN#KSTVEGIQASVK |
| 7 | P04908 | HLQLAIRN#DEN#ELNKLLGR |
| 8 | Q99798 | WVVIGDEN#YGEGSSREHAALEPR |
| 9 | P06733 | IGAEVYHN#LKNVIKEK |
| 10 | Q96KK5 | HLQLAIRN#DEELNKLLGK |
| 11 | P20290 | VQASLAAN#TFTITGHAETK |
| 12 | P26373 | VITEEEKN#FKAFASLR |
| 13 | P31948 | AAALEFLN#RFEEAKR |
| 14 | Q9ULV4 | AIFLADGN#VFTTGFSR |
| 15 | P48643 | QMAEIAVN#AVLTVADMERR |
| 16 | P46977 | TILVDNN#TWN#NTHISR |
| 17 | P21796 | KLETAVN#LAWTAGNSNTR |
| 18 | P10809 | LVQDVAN#NTNEEAGDGTTTATVLAR |
| 19 | P11021 | AKFEELN#MDLFR |
| 20 | Q9Y5L4 | VQIAVAN#AQELLQR |
| 21 | P62241 | IIDVVYN#ASNNELVR |
| 22 | P04406 | LISWYDN#EFGYSNR |
| 23 | P35232 | AAELIAN#SLATAGDGLIELR |
| 24 | P60842 | KGVAIN#MVTEEDKR |
| 25 | P07437 | ISVYYN#EATGGKYVPR |
| 26 | P62937 | VKEGMN#IVEAMER |
| 27 | P08670 | FLEQQN#KILLAELEQLKGQGK |
| 28 | P19338 | TLVLSN#LSYSATEETLQEVFEK |
| 29 | P58876 | AMGIMN#SFVNDIFER |
| 30 | O15160 | VLVYN#N#TSIVQDEILAHR |
| 31 | P04406 | VIHDN#FGIVEGLMTTVHAITATQK |
| 32 | P62910 | FLVHN#VKELEVLLMCNK |
| 33 | P09382 | SFVLN#LGKDSNNLCLHFNPR |
| 34 | Q8NE71 | IGFFN#QQYAEQLR |
| 35 | P50990 | ALAEN#SGVKANEVISK |
| 36 | P49411 | GITIN#AAHVEYSTAAR |
| 37 | P78371 | ILIAN#TGMDTDKIKIFGSR |
| 38 | P06733 | LAKYN#QLLRIEEELGSK |
| 39 | P07910 | VFIGN#LNTLVVKK |
| 40 | P40227 | VLAQN#SGFDLQETLVK |
| 41 | P62805 | VFLEN#VIRDAVTYTEHAK |
| 42 | P50990 | LFVTN#DAATILRELEVQHPAAK |
| 43 | P62805 | VFLEN#VIRDAVTYTEHAKR |
| 44 | P07910 | VFIGN#LNTLVVK |
| 45 | P04406 | VIHDN#FGIVEGLMTTVHAITATQKTVDGPSGKLWR |
| 46 | P04406 | VIHDN#FGIVEGLMTTVHAITATQKTVDGPSGK |
| 47 | P60174 | VIADN#VKDWSKVVLAYEPVWAIGTGK |
| 48 | P04406 | VIHDN#FGIVEGLMTTVHAITATQK |
| 49 | P68104 | THIN#IVVIGHVDSGKSTTTGHLIYK |
| 50 | P62899 | SAIN#EVVTREYTINIHKR |
| 51 | O75083 | N#IDN#PALADIYTEHAHQVVVAK |
| 52 | P14625 | LIIN#SLYKNKEIFLR |
| 53 | P45880 | DIFN#KGFGFGLVKLDVK |
| 54 | P46060 | VIN#LN#DNTFTEKGAVAMAETLK |
| 55 | P11047 | LLN#N#LTSIKIR |
| 56 | Q96AG4 | LVN#LQHLDLLN#NKLVTLPVSFAQLK |
| 57 | P11142 | IIN#EPTAAAIAYGLDKKVGAER |
| 58 | P14618 | IEN#HEGVRRFDEILEASDGIMVAR |
| 59 | P17987 | SQN#VMAAASIANIVK |
| 60 | Q99832 | MMPTPVILLKEGTDSSQGIPQLVSN#ISACQVIAEAVR |
| 61 | P06748 | TVSLGAGAKDELHIVEAEAMN#YEGSPIKVTLATLK |
| 62 | P06733 | AAVPSGASTGIYEALELRDN#DKTR |
| 63 | Q99832 | IN#ALTAASEAACLIVSVDETIKNPR |
| 64 | P14618 | LN#FSHGTHEYHAETIKNVR |
| 65 | P14618 | LN#FSHGTHEYHAETIK |
| 66 | P68371 | IN#VYYNEATGGKYVPR |
| 67 | P06733 | YN#QLLRIEEELGSKAK |
| 68 | P07900;  P08238 | TLTIVDTGIGMTKADLINN#LGTIAK |
| 69 | P09651 | GFGFVTYATVEEVDAAMN#ARPHKVDGR |
| 70 | P35232 | AAIISAEGDSKAAELIAN#SLATAGDGLIELR |
| 71 | P07737 | TLVLLMGKEGVHGGLIN#KK |
| 72 | P63244 | FVGHTKDVLSVAFSSDN#RQIVSGSR |
| 73 | Q13162 | GLFIIDDKGILRQITLN#DLPVGR |
| 74 | P60709 | GILTLKYPIEHGIVTN#WDDMEKIWHHTFYNELR |
| 75 | P68032 | GILTLKYPIEHGIITN#WDDMEKIWHHTFYNELR |
| 76 | P08238 | SIYYITGESKEQVAN#SAFVERVR |
| 77 | P08238 | SIYYITGESKEQVAN#SAFVER |
| 78 | P11142 | MKEIAEAYLGKTVTN#AVVTVPAYFNDSQR |
| 79 | P50454 | HLAGLGLTEAIDKN#KADLSR |
| 80 | P51991 | IFVGGIKEDTEEYN#LRDYFEK |
| 81 | A8TX70 | DVTIFSVGVYNAN#RSQLEEISGDSSLVFHVEN#FDHLKALER |
| 82 | P16403 | KALAAAGYDVEKN#NSR |
| 83 | P14625 | AQAYQTGKDISTN#YYASQKK |
| 84 | P61247 | GRVFEVSLADLQN#DEVAFRK |
| 85 | P16403 | ALAAAGYDVEKN#NSR |
| 86 | O43242 | AIRDGVIEASIN#HEKGYVQSK |
| 87 | P0CG48 | QLEDGRTLSDYN#IQKESTLHLVLR |
| 88 | P68371;  P07437 | MSMKEVDEQMLN#VQNKNSSYFVEWIPNNVK |
| 89 | O14818 | ALLEVVQSGGKN#IELAVMRR |
| 90 | P62937 | HTGPGILSMAN#AGPN#TN#GSQFFICTAKTEWLDGKHVVFGK |
| 91 | P63104 | SVTEQGAELSN#EERN#LLSVAYKNVVGAR |
| 92 | P23528 | LTGIKHELQAN#CYEEVKDR |
| 93 | P62937 | SIYGEKFEDEN#FILK |
| 94 | O43175 | SATKVTADVIN#AAEKLQVVGR |
| 95 | P07355 | TKGVDEVTIVN#ILTNR |
| 96 | P27348 | AVTEQGAELSN#EERNLLSVAYK |
| 97 | P61247 | VFEVSLADLQN#DEVAFRK |
| 98 | O75439 | AVEILADIIQN#STLGEAEIERER |
| 99 | P60709 | YPIEHGIVTN#WDDMEKIWHHTFYNELR |
| 100 | P26599 | GQPIYIQFSN#HKELKTDSSPNQAR |
| 101 | P30101 | KFLDAGHKLN#FAVASR |
| 102 | P08238 | IMKAQALRDN#STMGYMMAK |
| 103 | Q969G5 | AAQVQRLEAN#HGLLVAR |
| 104 | P39023 | ERLEQQVPVN#QVFGQDEMIDVIGVTK |
| 105 | P68032 | YPIEHGIITN#WDDMEKIWHHTFYNELR |
| 106 | P58876 | AMGIMN#SFVNDIFER |
| 107 | P22626 | N#MGGPYGGGN#YGPGGSGGSGGYGGR |
| 108 | P68363 | AVCMLSN#TTAIAEAWAR |
| 109 | Q9BQE3 | AVCMLSN#TTAVAEAWAR |
| 110 | P68363 | AVCMLSN#TTAIAEAWAR |
| 111 | P60709 | FRCPEALFQPSFLGMESCGIHETTFN#SIMK |
| 112 | P68371;  P07437 | MREIVHIQAGQCGN#QIGAK |
| 113 | P60709 | CDVDIRKDLYAN#TVLSGGTTMYPGIADR |
| 114 | P08708 | LLDFGSLSN#LQVTQPTVGMNFKTPR |
| 115 | Q13243 | NAPPVRTEN#RLIVENLSSR |
| 116 | P30101 | FLDAGHKLN#FAVASR |
| 117 | Q92945 | GGENVKAIN#QQTGAFVEISR |
| 118 | P62258 | YLAEFATGN#DRKEAAENSLVAYK |
| 119 | P13591 | DGQLLPSSN#YSNIK |
| 120 | P60891 | VTSIADRLN#VDFALIHKER |
| 121 | P39023 | AHLMEIQVN#GGTVAEKLDWAR |
| 122 | Q00341 | INIPPPSVN#RTEIVFTGEKEQLAQAVAR |
| 123 | Q8TAT6 | HVDNIMFEN#HTVADRFLDFWR |
| 124 | P08708 | LLDFGSLSN#LQVTQPTVGMNFKTPR |
| 125 | Q07065 | ERDFTSLEN#TVEERLTELTK |
| 126 | P78371 | MLPTIIADN#AGYDSADLVAQLR |
| 127 | P08708 | LLDFGSLSN#LQVTQPTVGMNFK |
| 128 | P10809 | LVQDVANN#TN#EEAGDGTTTATVLAR |
| 129 | P39023 | LEQQVPVN#QVFGQDEMIDVIGVTK |
| 130 | O43847 | ALNKGDAN#SEVTVYYQSGTR |
| 131 | Q99798 | WVVIGDEN#YGEGSSREHAALEPR |
| 132 | P08238 | VVVITKHN#DDEQYAWESSAGGSFTVR |
| 133 | Q99832 | TATQLAVN#KIKEIAVTVK |
| 134 | Q9Y4L1 | LKTVLSAN#ADHMAQIEGLMDDVDFKAK |
| 135 | Q00341 | MVADLVEN#SYSISVPIFKQFHK |
| 136 | P07437 | MAVTFIGN#STAIQELFKR |
| 137 | P48643 | QMAEIAVN#AVLTVADMERR |
| 138 | P27797 | SGTIFDN#FLITN#DEAYAEEFGNETWGVTK |
| 139 | P08238 | AQALRDN#STMGYMMAKK |
| 140 | O43175 | KGILVMN#TPNGNSLSAAELTCGMIMCLAR |
| 141 | P61978 | ALRTDYN#ASVSVPDSSGPER |
| 142 | O43175 | VTADVIN#AAEKLQVVGR |
| 143 | P11142 | ARFEELN#ADLFRGTLDPVEK |
| 144 | Q9NY33 | KLIVQPN#TRLEGSDVQLLEYEASAAGLIR |
| 145 | O60701 | VLDGLHN#ELQTIGFQIETIGK |
| 146 | P17987 | SLLVIPN#TLAVNAAQDSTDLVAKLR |
| 147 | P48643 | TSLGPN#GLDKMMVDKDGDVTVTN#DGATILSMMDVDHQIAK |
| 148 | Q13347 | TERPVN#SAALSPN#YDHVVLGGGQEAMDVTTTSTR |
| 149 | Q9Y490 | AVAAGN#SCRQEDVIATANLSRR |
| 150 | P13639 | GVQYLN#EIKDSVVAGFQWATKEGALCEENMR |
| 151 | P27708 | VLSEPN#PRPVFGICLGHQLLALAIGAK |
| 152 | P05386 | ALANVN#IGSLICN#VGAGGPAPAAGAAPAGGPAPSTAAAPAEEKKVEAK |
| 153 | Q9C0J9 | IIALQN#GER |
| 154 | P49411 | DKPHVN#VGTIGHVDHGKTTLTAAITK |
| 155 | Q9Y4L1 | VFGSQN#LTTVK |
| 156 | P20700 | SLETEN#SALQLQVTEREEVRGR |
| 157 | P07237 | MDSTAN#EVEAVKVHSFPTLK |
| 158 | C9JRZ8 | ALGVSN#FNHFQIER |
| 159 | P0CG48 | TLSDYN#IQKESTLHLVLR |
| 160 | Q04917 | AVTELN#EPLSNEDRNLLSVAYKNVVGAR |
| 161 | Q9Y4L1 | TVLSAN#ADHMAQIEGLMDDVDFKAK |
| 162 | Q04837 | QVEGKN#PVTIFSLATNEMWR |
| 163 | P36542 | MTAMDN#ASKNASEMIDKLTLTFNR |
| 164 | P58876 | AMGIMN#SFVNDIFERIAGEASR |
| 165 | P62081 | IVKPN#GEKPDEFESGISQALLELEMN#SDLKAQLR |
| 166 | P21796 | VTQSN#FAVGYKTDEFQLHTN#VNDGTEFGGSIYQKVNK |
| 167 | Q13586 | LAVTN#TTMTGTVLK |
| 168 | P04406 | VIHDN#FGIVEGLMTTVHAITATQK |
| 169 | P04406 | VIHDN#FGIVEGLMTTVHAITATQKTVDGPSGKLWR |
| 170 | P62241 | TLVKN#CIVLIDSTPYRQWYESHYALPLGR |
| 171 | P14618 | ITLDN#AYMEKCDEN#ILWLDYKNICK |
| 172 | P13929 | LAQSN#GWGVMVSHR |
| 173 | P51148 | QASPN#IVIALAGNKADLASKR |
| 174 | Q8WWM7 | NVDFN#YATKDKFTDSAIAMNSK |
| 175 | P46777 | TTTGN#KVFGALKGAVDGGLSIPHSTK |
| 176 | P18621 | NAESN#AELKGLDVDSLVIEHIQVNKAPK |
| 177 | P50990 | LFVTN#DAATILRELEVQHPAAK |
| 178 | P60174 | VIADN#VKDWSKVVLAYEPVWAIGTGK |
| 179 | P04406 | VIHDN#FGIVEGLMTTVHAITATQK |
| 180 | P15170 | SFVPN#MIGGASQADLAVLVISAR |
| 181 | P50454 | SLSN#STARN#VTWK |
| 182 | P50454 | SLSN#STARN#VTWKLGSR |
| 183 | P39023 | LIKN#N#ASTDYDLSDKSIN#PLGGFVHYGEVTNDFVMLK |
| 184 | P13929 | FGAN#AILGVSLAVCKAGAAEKGVPLYR |
| 185 | Q13263 | VLVN#DAQKVTEGQQERLER |
| 186 | P68104 | THIN#IVVIGHVDSGKSTTTGHLIYK |
| 187 | Q13813 | ETEN#VKSSEEIESAFR |
| 188 | O95302 | YHYN#GTFLDGTLFDSSHNR |
| 189 | Q99832 | AIKN#DSVVAGGGAIEMELSKYLR |
| 190 | P55072 | GFGSFRFPSGNQGGAGPSQGSGGGTGGSVYTEDN#DDDLYG |
| 191 | P61978 | HESGASIKIDEPLEGSEDRIITITGTQDQIQN#AQYLLQN#SVK |
| 192 | P20290 | VQASLAANTFTITGHAETKQLTEMLPSILN#QLGADSLTSLRR |
| 193 | P10809 | TLN#DELEIIEGMKFDRGYISPYFIN#TSK |
| 194 | Q13162 | SIN#TEVVACSVDSQFTHLAWIN#TPRR |
| 195 | Q13162 | SIN#TEVVACSVDSQFTHLAWINTPR |
| 196 | P02545 | NSN#LVGAAHEELQQSR |
| 197 | P11142 | IIN#EPTAAAIAYGLDKKVGAER |
| 198 | Q70UQ0 | ISN#LTIVQAEIK |
| 199 | Q9UNZ2 | SPN#ELVDDLFKGAKEHGAVAVER |
| 200 | P14618 | IEN#HEGVRRFDEILEASDGIMVAR |
| 201 | O75821 | VTN#LSEDTRETDLQELFRPFGSISR |
| 202 | P46060 | VIN#LNDNTFTEKGAVAMAETLKTLR |
| 203 | Q15365 | IAN#PVEGSSGRQVTITGSAASISLAQYLINAR |
| 204 | Q9BQE3 | IHFPLATYAPVISAEKAYHEQLTVAEITN#ACFEPAN#QMVK |
| 205 | Q9BQE3 | IHFPLATYAPVISAEKAYHEQLTVAEITN#ACFEPANQMVK |
| 206 | A8TX70 | GVSGEPGNPGPTGTLGAEGLQGPQGSQGN#PGR |
| 207 | Q9HCN8 | LHSHDIKYGSGSGQQSVTGVEASDDAN#SYWR |
| 208 | P62937 | SIYGEKFEDENFILKHTGPGILSMAN#AGPN#TN#GSQFFICTAK |
| 209 | P62937 | SIYGEKFEDENFILKHTGPGILSMAN#AGPN#TNGSQFFICTAK |
| 210 | P28072 | SGSAADTQAVADAVTYQLGFHSIELN#EPPLVHTAASLFK |
| 211 | P84243 | FQSAAIGALQEASEAYLVGLFEDTN#LCAIHAKR |
| 212 | Q71DI3 | FQSSAVMALQEASEAYLVGLFEDTN#LCAIHAKR |
| 213 | P84243 | FQSAAIGALQEASEAYLVGLFEDTN#LCAIHAK |
| 214 | Q71DI3 | FQSSAVMALQEASEAYLVGLFEDTN#LCAIHAK |
| 215 | Q7Z6Z7 | SAATSGAGSTTSGVVSGSLGSREIN#YILR |
| 216 | P14625 | EGSRTDDEVVQREEEAIQLDGLN#ASQIR |
| 217 | P78371 | LGGSLADSYLDEGFLLDKKIGVN#QPK |
| 218 | P48643 | TSLGPNGLDKMMVDKDGDVTVTN#DGATILSMMDVDHQIAK |
| 219 | Q15019 | TMLITHMQDLQEVTQDLHYEN#FRSER |
| 220 | P06748 | TVSLGAGAKDELHIVEAEAMN#YEGSPIKVTLATLK |
| 221 | P10809 | KPLVIIAEDVDGEALSTLVLN#R |
| 222 | P09622 | STDRVLGAHILGPGAGEMVN#EAALALEYGASCEDIAR |
| 223 | Q16576 | LHTFESHKDEIFQVHWSPHN#ETILASSGTDR |
| 224 | P14625 | NLLHVTDTGVGMTREELVKN#LGTIAK |
| 225 | P06576 | IGLFGGAGVGKTVLIMELIN#NVAK |
| 226 | P35232 | VFESIGKFGLALAVAGGVVN#SALYNVDAGHR |
| 227 | P13929 | VN#QIGSVTESIQACKLAQSN#GWGVMVSHR |
| 228 | P06733 | VN#QIGSVTESLQACKLAQANGWGVMVSHR |
| 229 | Q99832 | IN#ALTAASEAACLIVSVDETIKNPR |
| 230 | P14618 | LN#FSHGTHEYHAETIKNVR |
| 231 | P36578 | KN#NRQPYAVSELAGHQTSAESWGTGR |
| 232 | P06733 | YN#QLLRIEEELGSKAK |
| 233 | P52272 | IN#EILSNALKRGEIIAK |
| 234 | Q9Y265 | IN#GKDSIEKEHVEEISELFYDAK |
| 235 | P0CG48 | TLTGKTITLEVEPSDTIEN#VKAK |
| 236 | P19338 | IVTDRETGSSKGFGFVDFN#SEEDAK |
| 237 | P78371 | GMDKILLSSGRDASLMVTN#DGATILK |
| 238 | Q9NR12 | AAQAGVAVGDWVLSIDGEN#AGSLTHIEAQNKIR |
| 239 | Q15365 | QVTITGSAASISLAQYLIN#AR |
| 240 | P78371 | RQVLLSAAEAAEVILRVDN#IIK |
| 241 | Q15366 | AFAMIIDKLEEDISSSMTN#STAASRPPVTLR |
| 242 | P62805 | ISGLIYEETRGVLKVFLEN#VIR |
| 243 | P43686 | IQSIPLVIGQFLEAVDQN#TAIVGSTTGSN#YYVR |
| 244 | Q99798 | FRGHLDNISNNLLIGAIN#IEN#GKANSVR |
| 245 | P08238;  P07900 | TLTLVDTGIGMTKADLIN#N#LGTIAK |
| 246 | P63244 | RFVGHTKDVLSVAFSSDN#R |
| 247 | P07237 | QLAPIWDKLGETYKDHEN#IVIAK |
| 248 | P06576 | TREGNDLYHEMIESGVIN#LKDATSK |
| 249 | P11142 | NTTIPTKQTQTFTTYSDN#QPGVLIQVYEGER |
| 250 | P09651 | GFGFVTYATVEEVDAAMN#ARPHKVDGR |
| 251 | P08238;  P07900 | TLTLVDTGIGMTKADLIN#NLGTIAK |
| 252 | Q9Y3B8 | ALDDISESIKELQFYRNN#IFK |
| 253 | P35232 | AAIISAEGDSKAAELIAN#SLATAGDGLIELR |
| 254 | P43686 | IQSIPLVIGQFLEAVDQN#TAIVGSTTGSNYYVR |
| 255 | O75390 | AALPSHVVTMLDNFPTN#LHPMSQLSAAVTALNSESNFAR |
| 256 | Q01082 | LAEISDVWEEMKTTLKN#R |
| 257 | Q9BSJ8 | LTPRPTAAELEEVLQVN#SLIQTQK |
| 258 | Q13162 | GLFIIDDKGILRQITLN#DLPVGR |
| 259 | P04632 | ILGGVISAISEAAAQYN#PEPPPPR |
| 260 | P34897 | ISATSIFFESMPYKLN#PKTGLIDYN#QLALTAR |
| 261 | P68032 | GILTLKYPIEHGIITN#WDDMEKIWHHTFYNELR |
| 262 | P60709 | GILTLKYPIEHGIVTN#WDDMEKIWHHTFYNELR |
| 263 | P62701 | ERHPGSFDVVHVKDAN#GNSFATR |
| 264 | P20700 | LYKEELEQTYHAKLEN#AR |
| 265 | P08670 | TLLIKTVETRDGQVIN#ETSQHHDDLE |
| 266 | Q13347 | TFRTERPVNSAALSPN#YDHVVLGGGQEAMDVTTTSTR |
| 267 | P47756 | STLNEIYFGKTKDIVN#GLR |
| 268 | Q9H307 | LLEQKVELAQLQEEWN#EHNAK |
| 269 | P68032 | GILTLKYPIEHGIITN#WDDMEKIWHHTFYNELR |
| 270 | P60709 | GILTLKYPIEHGIVTN#WDDMEKIWHHTFYNELR |
| 271 | P11142 | MKEIAEAYLGKTVTN#AVVTVPAYFN#DSQR |
| 272 | P78371 | ILLSSGRDASLMVTN#DGATILKN#IGVDNPAAK |
| 273 | P68371;  P07437 | MSMKEVDEQMLNVQN#KN#SSYFVEWIPNNVK |
| 274 | P62937 | HTGPGILSMANAGPN#TNGSQFFICTAKTEWLDGKHVVFGK |
| 275 | Q9UJZ1 | ATVLESEGTRESAIN#VAEGKK |
| 276 | P18206 | LGATAEKAAAVGTAN#KSTVEGIQASVK |
| 277 | P08238 | SIYYITGESKEQVAN#SAFVERVR |
| 278 | P08238 | SIYYITGESKEQVAN#SAFVER |
| 279 | P05387 | MRYVASYLLAALGGN#SSPSAKDIK |
| 280 | P11142 | STAGDTHLGGEDFDN#RMVNHFIAEFK |
| 281 | P11142 | MKEIAEAYLGKTVTN#AVVTVPAYFNDSQR |
| 282 | P05387 | MRYVASYLLAALGGN#SSPSAK |
| 283 | P08670 | LLQDSVDFSLADAIN#TEFKNTR |
| 284 | P07954 | VAALTGLPFVTAPN#KFEALAAHDALVELSGAMN#TTACSLMK |
| 285 | P43243 | LASLMNLGMSSSLN#QQGAHSALSSASTSSHN#LQSIFNIGSR |
| 286 | Q9UN86 | IIRYPDSHQLFVGN#LPHDIDEN#ELKEFFMSFGNVVELR |
| 287 | Q15365 | AFAMIIDKLEEDIN#SSMTN#STAASRPPVTLR |
| 288 | P55072 | AVANETGAFFFLIN#GPEIMSK |
| 289 | P50454 | HLAGLGLTEAIDKN#KADLSR |
| 290 | Q96K17 | KLAVNNIAGIEEVN#MIKDDGTVIHFNNPK |
| 291 | P51991 | IFVGGIKEDTEEYN#LRDYFEK |
| 292 | Q15365 | AFAMIIDKLEEDIN#SSMTNSTAASRPPVTLR |
| 293 | P62258 | VAGMDVELTVEERN#LLSVAYKNVIGAR |
| 294 | Q14204 | SIIMRENFIPTIVN#FSAEEISDAIREK |
| 295 | Q96QV6 | HLQLAIRNDEELN#KLLGGVTIAQGGVLPN#IQAVLLPK |
| 296 | P68371;  P07437 | TLKLTTPTYGDLN#HLVSATMSGVTTCLRFPGQLNADLR |
| 297 | P09382 | GEVAPDAKSFVLN#LGKDSNNLCLHFNPR |
| 298 | P68363 | AYHEQLSVAEITN#ACFEPANQMVK |
| 299 | P04075 | RLQSIGTENTEEN#RR |
| 300 | P14625 | AQAYQTGKDISTN#YYASQKK |
| 301 | P61247 | GRVFEVSLADLQN#DEVAFRK |
| 302 | P20290 | LGVNNISGIEEVN#MFTNQGTVIHFNNPK |
| 303 | Q14697 | VLLVLELQGLQKN#MTR |
| 304 | P11142 | EIAEAYLGKTVTN#AVVTVPAYFNDSQR |
| 305 | P49368 | MLLDPMGGIVMTN#DGNAILREIQVQHPAAK |
| 306 | P35232 | FGLALAVAGGVVN#SALYNVDAGHR |
| 307 | P13639 | STAISLFYELSEN#DLNFIKQSK |
| 308 | Q9Y4L1 | MAGLKVLQLINDN#TATALSYGVFR |
| 309 | P62495 | LVDISYGGENGFN#QAIELSTEVLSNVK |
| 310 | P68371;  P07437 | SGPFGQIFRPDN#FVFGQSGAGN#NWAKGHYTEGAELVDSVLDVVRK |
| 311 | P60842 | GIDVQQVSLVIN#YDLPTN#RENYIHR |
| 312 | Q9Y3I0 | LVMEEAPESYKN#VTDVVNTCHDAGISKK |
| 313 | O00410 | VIQSADSKTKEN#VNATENCISAVGK |
| 314 | P16403 | ALAAAGYDVEKN#NSR |
| 315 | P16615 | TVEEVLGHFGVN#ESTGLSLEQVKK |
| 316 | Q9H299 | IQYQLVDISQDN#ALRDEMR |
| 317 | P12270 | VLLMELEEARGN#HVIRDEEVSSADISSSSEVISQHLVSYR |
| 318 | P68371;  P07437 | MSMKEVDEQMLN#VQNKNSSYFVEWIPNNVK |
| 319 | P68371;  P07437 | SGPFGQIFRPDN#FVFGQSGAGNNWAK |
| 320 | P23396 | FVADGIFKAELN#EFLTR |
| 321 | Q9UJ14 | GLSGLTQVLLN#VLTLN#RNLSDSLAR |
| 322 | P62937 | HTGPGILSMAN#AGPN#TNGSQFFICTAKTEWLDGKHVVFGK |
| 323 | P09382 | SFVLNLGKDSN#NLCLHFNPR |
| 324 | P04075 | QLLLTADDRVN#PCIGGVILFHETLYQK |
| 325 | P08670 | TVETRDGQVIN#ETSQHHDDLE |
| 326 | Q13813 | HQAFEAELHAN#ADRIR |
| 327 | Q16629 | YGGETKVYVGN#LGTGAGKGELER |
| 328 | Q16181 | THMQDLKDVTN#NVHYENYR |
| 329 | P17844 | LMEEIMSEKEN#KTIVFVETK |
| 330 | P31946 | AVTEQGHELSN#EERNLLSVAYK |
| 331 | Q13283 | IRHVDAHATLN#DGVVVQVMGLLSNNNQALRR |
| 332 | P61247 | VFEVSLADLQN#DEVAFRK |
| 333 | O75821 | ADDNATIRVTN#LSEDTRETDLQELFRPFGSISR |
| 334 | O75746 | LATATFAGIEN#KFGLYLPK |
| 335 | O75439 | AVEILADIIQN#STLGEAEIERER |
| 336 | Q01105 | IPNFWVTTFVN#HPQVSALLGEEDEEALHYLTR |
| 337 | Q9Y4L1 | YSHDFNFHIN#YGDLGFLGPEDLRVFGSQN#LTTVK |
| 338 | P39023 | ERLEQQVPVN#QVFGQDEMIDVIGVTK |
| 339 | P60709 | YPIEHGIVTN#WDDMEKIWHHTFYNELR |
| 340 | P68371;  P07437 | LTTPTYGDLN#HLVSATMSGVTTCLRFPGQLN#ADLRK |
| 341 | P68371;  P07437 | LTTPTYGDLN#HLVSATMSGVTTCLRFPGQLNADLR |
| 342 | Q00610 | KFNALFAQGN#YSEAAK |
| 343 | P46977 | TILVDNNTWN#NTHISR |
| 344 | P10809 | LVQDVANNTN#EEAGDGTTTATVLAR |
| 345 | P31946 | YLSEVASGDN#KQTTVSNSQQAYQEAFEISKK |
| 346 | P0DMV9;  P11142 | VEIIANDQGN#RTTPSYVAFTDTERLIGDAAK |
| 347 | Q9Y4L1 | AVGKEELGKN#INADEAAAMGAVYQAAALSK |
| 348 | P39023 | ERLEQQVPVN#QVFGQDEMIDVIGVTK |
| 349 | P68032 | YPIEHGIITN#WDDMEKIWHHTFYNELR |
| 350 | P58876 | AMGIMNSFVN#DIFER |
| 351 | P14625;  P08238 | EIFLRELISN#ASDALDKIR |
| 352 | P58876 | AMGIMN#SFVNDIFERIAGEASR |
| 353 | P06733 | HIADLAGNSEVILPVPAFNVINGGSHAGNKLAMQEFMILPVGAAN#FR |
| 354 | Q9UHD8 | THMQN#IKDITSSIHFEAYR |
| 355 | P62937 | SIYGEKFEDENFILKHTGPGILSMAN#AGPN#TN#GSQFFICTAK |
| 356 | P62937 | SIYGEKFEDENFILKHTGPGILSMAN#AGPN#TNGSQFFICTAK |
| 357 | P06748 | TVSLGAGAKDELHIVEAEAMN#YEGSPIKVTLATLK |
| 358 | P14625 | IMKAQAYQTGKDISTN#YYASQK |
| 359 | P58876 | AMGIMN#SFVNDIFERIAGEASR |
| 360 | P68371;  P07437 | MSMKEVDEQMLN#VQN#KN#SSYFVEWIPNNVK |
| 361 | P34897 | AALEALGSCLN#NKYSEGYPGKR |
| 362 | P08865 | YVDIAIPCN#NKGAHSVGLMWWMLAR |
| 363 | P25786 | HMSEFMECNLN#ELVKHGLR |
| 364 | Q01082 | SWHNVYCVINN#QEMGFYKDAK |
| 365 | A6NHL2 | SICMLSN#TTAIVEAWAR |
| 366 | Q13151 | SRCFGFVTYSN#VEEADAAMAASPHAVDGN#TVELKR |
| 367 | P60709 | FRCPEALFQPSFLGMESCGIHETTFN#SIMK |
| 368 | P68371;  P07437 | LTTPTYGDLNHLVSATMSGVTTCLRFPGQLN#ADLRK |
| 369 | P60709;  Q6S8J3 | LCYVALDFEQEMATAASSSSLEKSYELPDGQVITIGN#ER |
| 370 | Q7KZF4 | TCATVTIGGIN#IAEALVSK |
| 371 | P68032 | LCYVALDFEN#EMATAASSSSLEKSYELPDGQVITIGNER |
| 372 | P06733 | SCNCLLLKVN#QIGSVTESLQACK |
| 373 | P25789 | LYQVEYAMEAIGHAGTCLGILAN#DGVLLAAERR |
| 374 | P13929 | VNQIGSVTESIQACKLAQSN#GWGVMVSHR |
| 375 | Q7Z7H5 | FTFTSHTPGDHQICLHSN#STR |
| 376 | O15372 | MDSLLIAGQINTYCQN#IKEFTAQNLGK |
| 377 | P68371;  P07437 | MREIVHIQAGQCGN#QIGAK |
| 378 | P14618 | ITLDNAYMEKCDEN#ILWLDYKNICK |
| 379 | P62266 | KITAFVPNDGCLN#FIEENDEVLVAGFGR |
| 380 | P13639 | YVEPIEDVPCGN#IVGLVGVDQFLVKTGTITTFEHAHN#MR |
| 381 | P37802 | N#FSDN#QLQEGKNVIGLQMGTNR |
| 382 | P14625 | N#LLHVTDTGVGMTREELVKN#LGTIAK |
| 383 | P51149 | N#N#IPYFETSAKEAINVEQAFQTIAR |
| 384 | P11142 | N#TTIPTKQTQTFTTYSDN#QPGVLIQVYEGER |
| 385 | P14625 | N#LLHVTDTGVGMTREELVK |
| 386 | P13639 | N#MSVIAHVDHGKSTLTDSLVCK |
| 387 | P13639 | N#MSVIAHVDHGKSTLTDSLVCKAGIIASAR |
| 388 | O15371 | N#LAMEATYINHNFSQQCLR |
| 389 | P14625 | N#LLHVTDTGVGMTREELVK |
| 390 | P36578 | N#IPGITLLNVSKLNILKN# |
| 391 | P51149 | N#NIPYFETSAKEAINVEQAFQTIAR |
| 392 | P07602 | N#YISQYSEIAIQMMMHMQPK |
| 393 | Q99733 | N#VDMLSELVQEYDEPILK |
